# Application of LpxC enzyme inhibitor to inhibit some fast-growing bacteria in human gut bacterial culturomics

**DOI:** 10.1186/s12866-019-1681-6

**Published:** 2019-12-30

**Authors:** Fengyi Hou, Yuxiao Chang, Zongyu Huang, Ni Han, Lei Bin, Huimin Deng, Zhengchao Li, Zhiyuan Pan, Lei Ding, Hong Gao, Ruifu Yang, Fachao Zhi, Yujing Bi

**Affiliations:** 1 Guangdong Provincial Key Laboratory of Gastroenterology, Institute of Gastroenterology of Guangdong Province, Department of Gastroenterology, Nanfang Hospital, Southern Medical University, Guangzhou, China; 2 State Key Laboratory of Pathogen and Biosecurity, Beijing Institute of Microbiology and Epidemiology, Beijing, China; 3 Shijitan Hospital, Capital Medical University, Beijing, China

**Keywords:** Culturomics, Human gut microbiota, CHIR-090, LpxC inhibitor

## Abstract

**Background:**

Culturomics can ascertain traces of microorganisms to be cultivated using different strategies and identified by matrix-assisted laser desorption/ionization–time-of-flight mass spectrometry or 16S rDNA sequencing. However, to cater to all requirements of microorganisms and isolate as many species as possible, multiple culture conditions must be used, imposing a heavy workload. In addition, the fast-growing bacteria (e.g., *Escherichia*) surpass the slow-growing bacteria in culture by occupying space and using up nutrients. Besides, some bacteria (e.g., *Pseudomonas*) suppress others by secreting antibacterial metabolites, making it difficult to isolate bacteria with lower competence. Applying inhibitors to restrain fast-growing bacteria is one method to cultivate more bacterial species from human feces.

**Results:**

We applied CHIR-090, an LpxC enzyme inhibitor that has antibacterial activity against most Gram-negative bacteria, to culturomics of human fresh feces. The antibacterial activity of CHIR-090 was first assessed on five Gram-negative species of bacteria (*Escherichia coli*, *Pseudomonas aeruginosa*, *Klebsiella pneumoniae*, *Proteus vulgaris*, and *Bacteroides vulgatus*), all of which are commonly isolated from the human gut. Then, we assessed suitable concentrations of the inhibitor. Finally, CHIR-090 was applied in blood culture bottles for bacterial cultivation. In total, 102 species from five samples were identified. Of these, we found one new species, two species not reported previously in the human gut, and 11 species not previously isolated from humans.

**Conclusions:**

CHIR-090 can suppress *E. coli*, *P. aeruginosa*, *K. pneumoniae*, *Pro. vulgaris*, but not *B. vulgatus.* Compared with the non-inhibitor group, CHIR-090 increased bacteria isolation by 23.50%, including four species not reported in humans and one new species. Application of LpxC enzyme inhibitor in culturomics increased the number of species isolated from the human gut.

## Background

Recent studies have revealed that the gut microbiota plays an important role in maintaining homeostasis and human health [1, 2]. The revolution of metagenomics has helped further our comprehension of the human gut microbiota, but it has left a large number of unidentified sequences that may belong to unknown species [3, 4]. The renaissance of culturomics is trying to fill these gaps by isolating and analyzing the pure culture of human gut microorganisms [5, 6]. To date, more than 1000 species of bacteria in the human gut have been cultivated and studied [7]. However, because culturomics requires multiple cultivation conditions to satisfy the preferences of different species in the microbiota, it is a time- and labor-consuming process, which has long hindered progress in this area [8]. In our work isolating gut microorganisms, we found that *Escherichia coli*, a rapidly and easily grown species, quickly occupied most of the space of a culture plate, making it more difficult to isolate other bacterial species. In addition, the culture diversity of fecal samples with large numbers of *Pseudomonas aeruginosa* is much lower than that of samples without *P. aeruginosa*. Therefore, we postulate that inhibiting the growth of *E. coli* and *P. aeruginosa* in a culture system could result in a more efficient isolation of previously unidentified species.

CHIR-090 is an inhibitor of the enzyme LpxC, the key enzyme of lipid A biosynthesis in Gram-negative bacteria [9]. When lipid A biosynthesis, a cytoderm lipid that shields Gram-negative bacteria, is suppressed, the cytomembrane of Gram-negative bacteria can easily crack, leading to the death of bacteria [10]. Previous studies have demonstrated that CHIR-090 shows substantial antibacterial activity against both *E. coli* and *P. aeruginosa* [11].

In this study, we evaluated the antibacterial or inhibitory capacity of CHIR-090 against five Gram-negative bacteria. Furthermore, we determined the optimal concentration of CHIR-090 in blood culture bottles for fecal microbiota enrichment before prolonged cultivation. Finally, we applied CHIR-090 in culturomics of five fecal samples from healthy adults to evaluate the feasibility of using CHIR-090 to increase isolation of previously unreported bacterial species.

## Results

### Antibacterial capacity of CHIR-090

To evaluate the ability of CHIR-090 to suppress the growth of five Gram-negative bacteria (*E. coli*, *P. aeruginosa*, *K. pneumoniae*, *Pro. vulgaris*, and *B. vulgatus*), CHIR-090 was added to culture medium. We found that not all the Gram-negative bacteria were inhibited by CHIR-090 and the effective concentrations differed by species (Table 1). *E. coli*, *P. aeruginosa*, *K. pneumoniae*, and *Pro. vulgaris* were completely suppressed by CHIR-090 but at different concentrations of the inhibitor. Growth of *Pro. vulgaris* was inhibited with 8 μg/mL CHIR-090, whereas growth of *E. coli* and *P. aeruginosa* was inhibited at 40 μg/mL CHIR-090. *K. pneumoniae* was not inhibited until the concentration of CHIR-090 reached 200 μg/mL. Finally, growth of *B. vulgatus* was not affected by CHIR-090, even at the highest concentration tested. These differences in antibacterial activity may be related to different coding sequences (Additional file 1: Table S1) of enzyme LpxC, which result in diverse structures of this enzyme that affect the tightness of the binding with CHIR-090 [12].

**Table 1:** Counts (colony-forming units, CFU) of five Gram-negative bacteria after co-culturing with different concentrations of CHIR-090

	Bacteria	CFU					
		Blank	DMSO	8 μg/mL	40 μg/mL	80 μg/mL	200 μg/mL
Sensitive	*Escherichia coli*	> 500	> 500	4	0	0	0
	*Pseudomonas aeruginosa*	> 500	> 500	46	0	0	0
	*Klebsiella pneumoniae*	> 500	> 500	> 500	> 500	26	0
	*Proteus vulgaris*	> 500	> 500	0	0	0	0
Insensitive	*Bacteroides vulgatus*	> 500	> 500	> 500	> 500	> 500	> 500

### Determination of optimal concentration of CHIR-090

In this study, we adopt Lagier’s group strategy [8]: we enriched fecal samples in blood culture bottles with 5% sheep blood and 5% rumen fluid and then subcultured the mixtures on YCFA plates, followed by subculture and identification of colonies. Although 200 μg/mL CHIR-090 could suppress 4 common fast-growing Gram-negative bacteria, we also tested CHIR-090 at 400 and 800 μg/mLl, because of the large numbers of microorganisms that the human gut harbors and the continuous consumption of the inhibitor during the prolonged enrichment of bacteria in our study. Because we observed that the effective concentration of CHIR-090 varied with bacterial species, we determined the optimal concentration before extending the experiment. To do so, fecal sample 1 (F1) was cultivated with CHIR-090 at 80, 400, and 800 μg/mL in blood culture bottles with 5% sheep blood and 5% rumen fluid; bottles with dimethyl sulphoxide (DMSO) and without treatment (blank) were used as controls. In this experiment, we isolated 19 species of bacteria in total. The percentages of colony-forming units of each bacterium under different CHIR-090 concentrations are shown in Additional file 1: Figure S1a, and that in anaerobic or aerobic conditions are shown in Additional file 1: Figure S1b and Additional file 1: Figure S1c, respectively. *E. coli* and *Enterococcus faecium* occupied the largest percentages in DMSO and blank bottles, in which only 6 and 5 species, respectively, were isolated. In the CHIR-090 bottles, growth of *E. coli* was suppressed, allowing the number of isolated species to increase to 12 species in the 80 and 400 μg/mL bottles, at equal percentages. However, when the concentration of CHIR-090 was increased to 800 μg/mL, the diversity decreased to 6 species; at 800 μg/mL, *Enterococcus faecalis* prevailed though no *E. coli* were found. Because CHIR-090 is a time-dependent inhibitor, we determined 400 μg/mL to be the optimal concentration for subsequent experiments.

### Evaluation of CHIR-090 in human gut culturomics

**Fig. 1:**
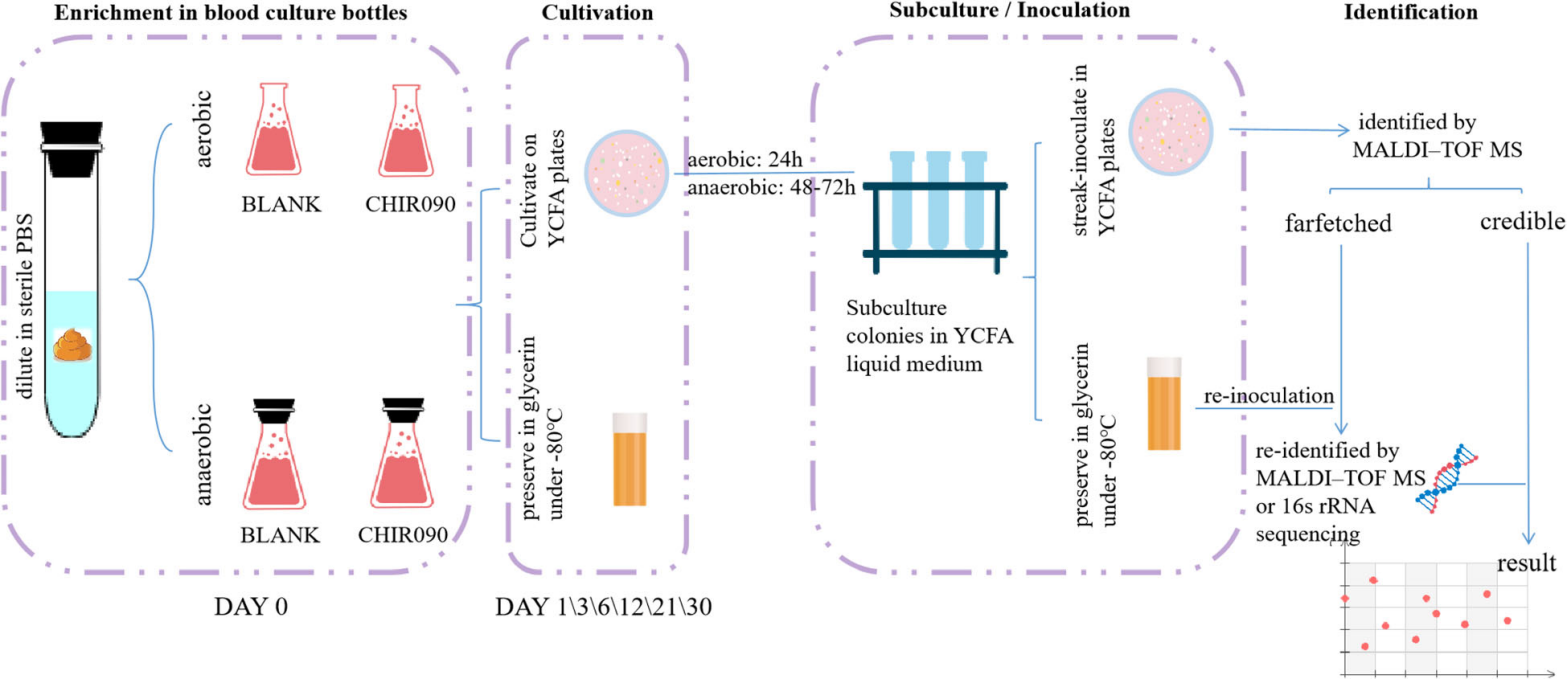
Workflow of the culturomics strategy. Fecal samples were enriched in blood culture bottles containing 5% sheep blood, 5% rumen fluid, and CHIR-090 for one month, plating subsamples at 1, 3, 6, 12, 21, and 30 days, subculturing the resulting colonies in YCFA liquid medium (all cultures were preserved in glycerin at − 80 °C or liquid nitrogen for the reuse of bacteria), streak-inoculating the subcultures, and finally identifying the species by MALDI–TOF MS or 16S rDNA sequencing

**Fig. 2:**
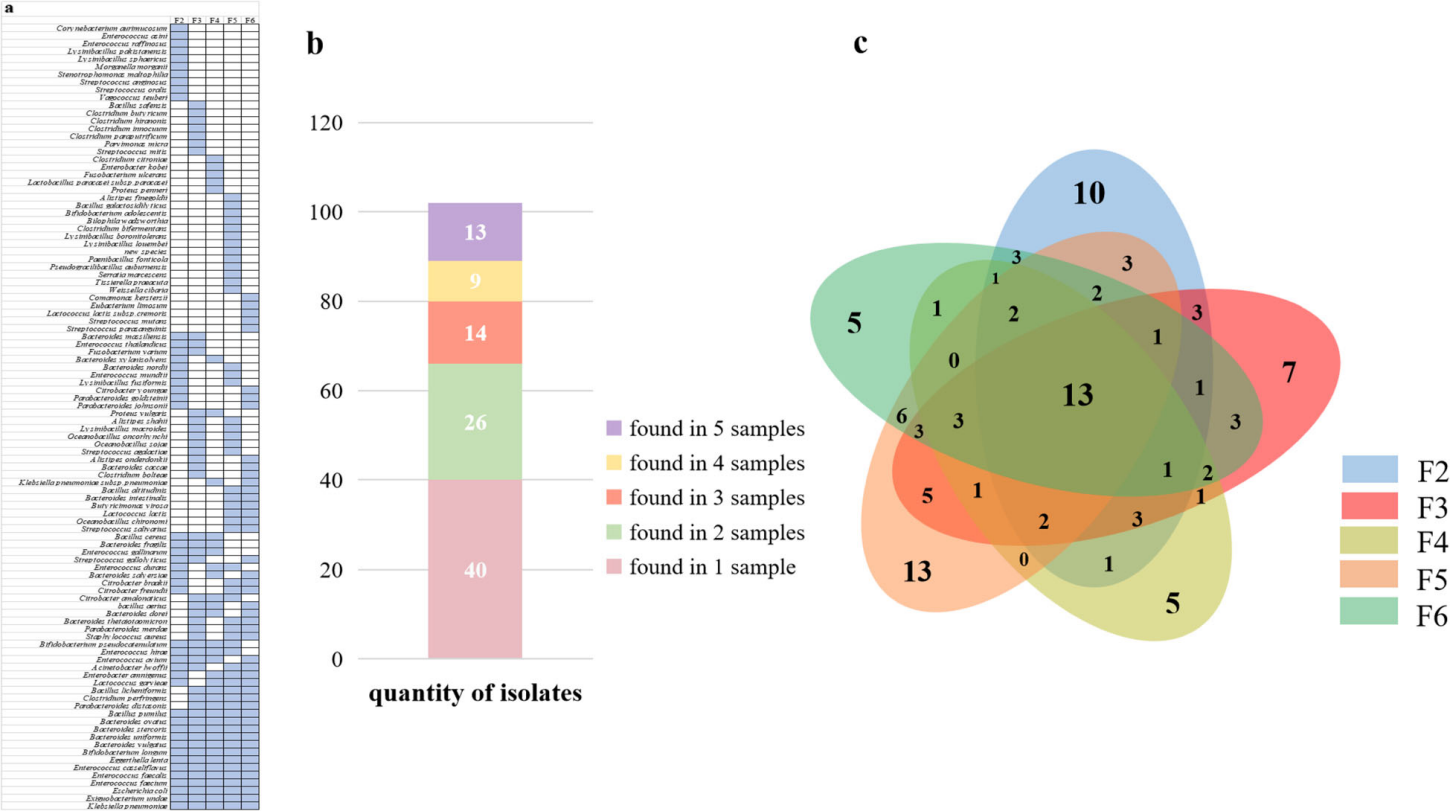
Overall information of 102 isolated species in five samples. **a** Distribution of 102 bacteria species in 5 samples. The light blue color represents the presence and blank space indicates the absence. **b** Numbers of bacterial species found in sample(s). **c** Amounts of overlap between and among samples

**Fig. 3:**
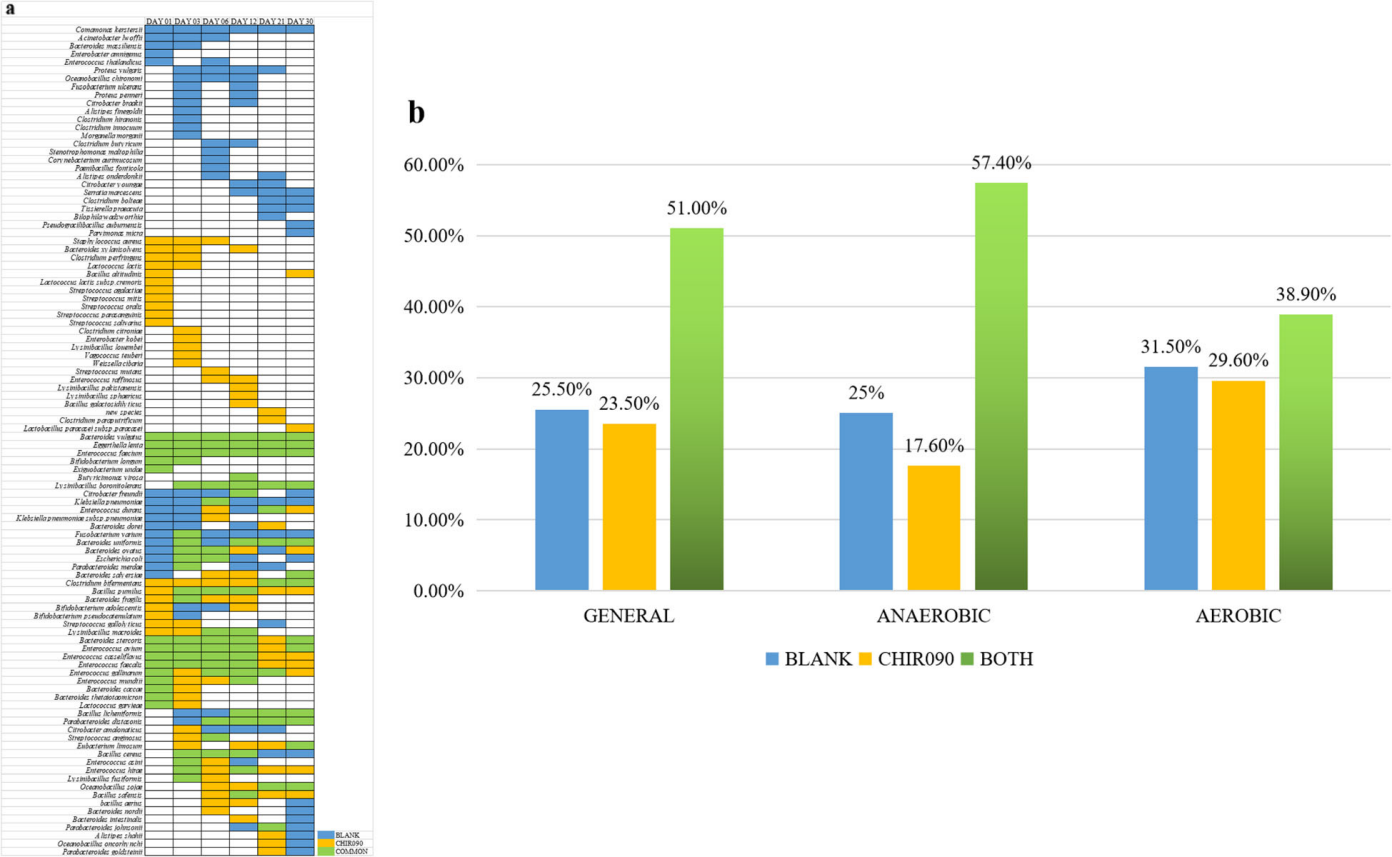
Distribution and percentages of bacteria in each group. **a** The distribution of 102 bacteria in different groups at 6 points in time under general condition. **b** The percentage of all bacteria identified in blank bottles and CHIR-090 bottles under general, anaerobic, and aerobic conditions. Blue represents the blank group, yellow represents the CHIR-090 group, and green indicates presence in both bottles (common group)

We enriched fecal samples for 1 month, plating subsamples at 1, 3, 6, 12, 21, and 30 days, subculturing the resulting colonies in YCFA liquid medium, streak-inoculating the subcultures, and finally identifying the species by Matrix-assisted laser desorption/ionization–time-of-flight mass spectrometry (MALDI–TOF MS) or 16S rDNA sequencing (Fig. 1). Overall, we identified 102 species from five fresh fecal samples (Fig. 2 a). Taxonomic information showed that the isolates covered five phyla: *Actinobacteria*, *Bacteroidetes*, *Firmicutes*, *Fusobacteria*, and *Proteobacteria* (Additional file 2: Table S2). Forty species were found only in 1 sample, 26 species were found in 2 samples, 14 species were found in 3 samples, 9 species were found in 4 samples, and 13 species were found in 5 samples (Fig. 2 b). The amount of overlap among samples is shown in Fig. 2 c. During the 1-month culture, different bacteria existed in different groups at different time points (Fig. 3 a), demonstrating that a prolonged culture time results in more complete isolation of species. The addition of CHIR-090 or not resulted in very different microecology, such that 23.5% of bacteria were only isolated in CHIR-090 bottles, 25.5% were only isolated from bottles without CHIR-090, and 51% were found in both bottles in general condition (including anaerobic and aerobic condition) (Fig. 3 b). Analysis of anaerobic or aerobic conditions respectively resulted in similar findings (Additional file 1: Figure S2a, Additional file 1: Figure S2b). The distributions of each species and percentages of each group in samples are shown in Additional file 1: Figure S3a-t. Moreover, addition of CHIR-090 accounted for about one-third of the bacterial isolates from one stool sample (Table 2), indicating that application of CHIR-090 results in discovery of more bacterial species and, to some degree, offsets the sample source deficiency. Among 102 species identified in our experiments, one isolate was a potentially novel species, two were not previously reported to be associated with the human gut, and 11 were not previously reported to be isolated from humans, four of which were from bottles with CHIR-090. The potentially novel bacterial species was also identified in a CHIR-090 bottle (Table 3). Eight of these species came from one sample and CHIR-090 accounted for five of them (Table 3).

**Table 2:** Percentages of bacteria present in blank or CHIR090 group in each sample under different conditions

	BA%	CHA%	CA%	BO%	CHO%	CO%	B%	CH%	C%
F2	35.70	35.70	28.60	42.30	34.60	23.10	37.80	28.90	33.30
F3	36.40	30.30	33.30	57.10	23.80	19.10	42.80	28.60	28.60
F4	25.90	40.70	33.30	35.30	35.30	29.40	25.00	30.60	44.40
F5	20.70	37.90	41.40	32.10	42.90	25.00	25.50	35.30	39.20
F6	25.80	38.70	35.50	63.20	21.10	15.80	39.10	30.40	30.40
Mean	28.90	36.60	34.40	46.00	31.50	22.48	34.04	30.76	25.18

*BA*, blank group, anaerobic, *CHA*, CHIR-090 group, anaerobic, *CA*, common group, anaerobic, *BO*, blank group, aerobic, *CHO*, CHIR-090 group, aerobic; CO, common group, aerobic, *B*, blank group, general, *CH*, CHIR-090 group, general, *C*, common group, general

**Table 3:** Fourteen bacterial species that have not previously been isolated from humans or the human gut

	Bacteria	Source	Group	Initial source
Not isolated in human	*new species (Bacillus.sp)*	F5	CHIR090	/
	*Bacillus altitudinis*	F5,6	CHIR090	cryogenic tubes [13]
	*Lysinibacillus louembei*	F5	CHIR090	alkaline fermented leaves of cassava [14]
	*Lysinibacillus pakistanensis*	F2	CHIR090	the Manasbal Lake [15]
	*Vagococcus teuberi*	F2	CHIR090	fermented Cow Milk [16]
	*Oceanobacillus chironomi*	F5,6	Blank	chironomid egg mass [17]
	*Pseudogracilibacillus auburnensis*	F5	Blank	rhizosphere of *Zea mays* [18]
	*Paenibacillus fonticola*	F5	Blank	warm spring [19]
	*Exiguobacterium undae*	F2,3,4,5,6	Common	pond water [20]
	*bacillus aerius*	F3,4,6	Common	cryogenic tubes [13]
	*Lysinibacillus macroides*	F3,5	Common	cow dung [21]
	*Bacillus safensis*	F3	Common	spacecraft and assembly-facility surfaces [22]
Not isolated in human gut	*Fusobacterium ulcerans*	F4	Blank	tropical ulcers [23]
	*Enterobacter amnigenus*	F2,4,5,6	Blank	blood of a heart transplant patient [24]

## Discussion

In recent decades, researchers have attempted to understand how the gut microbiome affects human health because it is the largest immune organ in the body [25–28]. Revolutions in sequencing techniques have added, little by little, to the landscape of the gut microbiome [29, 30]. However, this process has reached a plateau because sequencing techniques are limited in that they can only identify bacteria to the species level or, worse, can result in mismatched sequences [31]. Culturomics can identify bacteria to the strain level by applying multiple cultivation conditions to isolate the full diversity of the microbiota and by using MALDI–TOF MS or 16S rRNA amplification and sequencing for identification [32]. The isolates can be used in mechanistic studies, especially those that focus on interactions with other bacteria and with the host [33–35]. However, culturomics has its own limitations. First, to expand the culturable gut microbiota repertoire, complex conditions are essential to meet the various preferences of all species that make up the microbiota, which requires a great amount of work, although some studies have tried to simplify culture conditions [8]. This time- and labor-consuming workload has long hindered progress in culturomics. Second, interactions among bacteria increase the difficulty of isolation. For example, before slow-growing bacteria can grow sufficiently to be identified, *E. coli* have already dominated the culture and prevented further growth of lagging species. Moreover, an antibacterial peptide produced by *Pseudomonas* can affect the growth of other bacteria in co-culture [36]. Selected culture media can help distinguish bacteria, but selective media are generally used to isolate specific species and thus are not efficient for mass isolation [37, 38]. The use of phage is an option to kill *Escherichia* and *Pseudomonas*; however, the extreme specificity of phage to the strain level makes it a less effective option for stopping growth of various *Escherichia* and *Pseudomonas* species [39]. The use of antibacterial agents is another way to suppress fast-growing bacteria. CHIR-090 inhibits the enzyme LpxC, which catalyzes the first irreversible step of lipid A biosynthesis of *E. coli* and most Gram-negative bacteria, and aroused our interest [12]. Previous studies showed that CHIR-090 could inhibit the growth of *E. coli* and *P. aeruginosa* [9, 40]. In our studies, CHIR-090 excelled in suppressing growth of most Gram-negative bacteria that we studied, especially *E. coli*. Its ability to do so depends to a certain extent on the LpxC coding sequence, which determines the structure of LpxC. LpxC source of coding sequence on line may predict the effect of CHIR-090 before using it as an antibacterial agent [12].

Cultures with sheep blood and rumen fluid can satisfy a large majority of gut microorganisms [8]. In our previous studies (unpublished data) we found that prolonging the culture time can isolate more bacteria because different bacteria exist at different times over a month. We also found that more bacteria are isolated early in the 1-month culture period; however, after day 10, the microbiota varies less. Prolonging enrichment over 30 days is not recommended. Therefore, we chose to subculture on days 1, 3, 6, 12, 21, and 30. Fecal culture represents a complicated microbial ecology in that it varies throughout a 1-month enrichment. CHIR-090 is the main factor affecting microbial ecology because it inhibits growth of many of the Gram-negative bacteria (40% of Gram-negative bacteria are inhibited in this study), thus disturbing the microbiota. Meanwhile, it allows other bacteria to grow because space and nutrients are spared. Thus, without altering other conditions and adding CHIR-090 into blood culture bottles, the number of species isolated can be increased by at least a quarter. Increasing the number of samples would allow isolation of more bacteria; however, when a source is difficult to obtain, CHIR-090 can, to some degree, offset this deficiency. Therefore, we believe that CHIR-090 has great potential for isolating diverse species in the gut microbiota and discovering new bacterial species.

## Conclusion

In this study, we introduced CHIR-090 in culturomics of human gut microbiota. First, we found that CHIR-090 could inhibit *E. coli, P. aeruginosa, K. pneumoniae,* and *Pro. vulgaris,*which are all commonly isolated species in human feces. Second we optimize the concentration of CHIR-090 in blood culture bottles for human feces culturing. Under the concentration of 400 μg/mL, CHIR-090 increased bacterial diverisity of isolates in five fecal samples by 23.50%, showing its usefulness in fecal microbiota culturomics. Application of LpxC enzyme inhibitor increased the number of species isolated from the human gut.

## Methods

### Bacterial strains

*E. coli*, *P. aeruginosa*, *K. pneumoniae*, *Pro. vulgaris*, and *B. vulgatus* were isolated from the human feces and preserved at − 80 °C in our laboratory. MALDI–TOF MS was conducted by QuantiHealth Technology Co. Ltd. (Beijing, China) to confirm the species identity of the inoculated colonies before usage.

### CHIR-090

CHIR-090 (Beijing BioRab Technology Co. Ltd., Beijing, China), also called benzamide, *N*-[(1S,2R)-2-hydroxy-1-[(hydroxyamino)carbonyl] propyl]-4-[2-[4- (4-morpholinylmethyl) phenyl]ethynyl], is a two-step, slow, tight-binding inhibitor of *E. coli* LpxC. Before usage, CHIR-090 was dissolved in DMSO.

### Antibacterial capacity of CHIR-090

Colonies of *E. coli*, *P. aeruginosa*, *K. pneumoniae*, *Pro. vulgaris*, and *B. vulgatus* were inoculated into YCFA liquid medium [41] with different concentrations of CHIR-090 (8, 40, 80, and 200 μg/mL). For controls, we inoculated colonies into bottles with DMSO and into bottles without any treatment (“blanks”). All bottles were incubated at 37 °C under aerobic conditions for 24 h. The cultures were then cultivated using the agar dilution method at 37 °C under aerobic conditions for 24 h. Finally, colony-forming units (CFU) on each agar plate were counted.

### Stool samples and pretreatment

Six fresh fecal samples were collected from six healthy human adults who met the screening criteria for donors in the European Fecal Microbiota Transplantation (FMT) criteria [42] and were designated F1 to F6. Each fecal sample (500 mg) was diluted in 15 ml of sterile phosphate buffer saline (PBS) immediately after collection and blended thoroughly. Then, 0.5 ml of each sample suspension was injected into a blood culture bottle with 5% sheep blood and 5% rumen liquid for enrichment.

### Optimization of the applied concentration of CHIR-090

Dissolved CHIR-090 was injected into blood culture bottles (with 5% sheep blood and 5% rumen fluid) to reach a concentration of 80, 400, or 800 μg/ml; bottles with DMSO and without treatments (blanks) were used as controls. After diluting 500 mg of fresh fecal sample F1 with 15 ml of sterile PBS, 0.5 ml of the diluted suspension was added into each prepared bottle (liquid volume 50 ml) and incubated at 37 °C, under anaerobic or aerobic condition for 24 h. The cultures were then cultivated using the agar dilution method at 37 °C under aerobic conditions for 24 h and anaerobic conditions for 48 h. The colonies were collected and identified by MALDI–TOF MS; if colonies were not identifiable, they were subjected to 16S rDNA sequencing.

### Cultivation strategy

Blood cultures containing 5% sheep blood, 5% rumen fluid, and CHIR-090 (400 μg/ml) were used to enrich the fresh stool dilutions at 37 °C under anaerobic and aerobic conditions for 1 month. On days 1, 3, 6, 12, 21, and 30, samples of enriched cultures were extracted from the bottles by syringe, and doubling dilutions were spread onto YCFA plates for culture at 37 °C under aerobic conditions for 24 h or anaerobic conditions for 48 h. Colonies were picked according to their appearance (size, color, and shape) for subculture in YCFA liquid medium. The subcultures were then streak-inoculated and later identified by MALDI–TOF MS or 16S rDNA sequencing.

### MALDI–TOF MS

Colonies were first identified by MALDI–TOF MS using an Autof ms1000 system (QuantiHealth Technology Co. Ltd., Beijing, China) after being deposited into 1 ml of lysis buffer (70% formic acid) and 1 ml of matrix solution (saturated α-cyano acid-4-hydroxycinnamic in 50% acetonitrile and 2.5% trifluoroacetic acid). Each spectrum was compared with those of known samples in the database. A colony was not labeled as credible at the species level without a total score ≥ 9.0.

### 16S rDNA sequencing

Colonies that were not identified by MALDI-TOF MS were subjected to 16S rDNA sequencing with primers 27F (5′-AGAGTTTGATCMTGGCTCAG-3′) and 1492R (5′-GGTTACCTTGTTACGACTT-3′) (Tsingke Biological Technology Co. Ltd., Beijing, China). For identification at the species level, we chose a threshold similarity of > 98.0%. An isolate with a similarity value below this threshold was suspected to be a new species.

### Classification of cultivated species

We used an online database of isolated bacteria in humans (http://hpr.mediterranee-infection.com/arkotheque/client/ihu_bacteries/recherche/index.php) to classify all isolates into four categories: new species, known species in human gut, species previously isolated from the environment but first isolated from humans, and species previously isolated from humans but first isolated from human gut. We also conducted literature searches on PubMed to compare against published papers and confirm the classification.

## Supplementary information

**Additional file 1:** Supplementary table and figure.

**Additional file 2: Table S2** Taxonomic information of 102 species of bacteria.

## Data Availability

All data generated or analyzed during current study are available from the corresponding author on reasonable request.

## Abbreviations

***B. vulgatus***: *Bacteroides vulgatus*
**CFU**: Colony-forming units
**DMSO**: Dimethyl sulphoxide
***E. coli***: *Escherichia coli*
**FMT**: Fecal microbiota transplantation
***K. pneumoniare***: *Klebsiella pneumoniae*
**MALDI–TOF MS**: Matrix-assisted laser desorption/ionization–time-of-flight mass spectrometry
***P. aeruginosa***: *Pseudomonas aeruginosa*
**PBS**: Phosphate buffer saline
***Pro. vulgaris***: *Proteus vulgaris*
