# Supplementary material for: Application of LpxC enzyme inhibitor to inhibit some fast-growing bacteria in human gut bacterial culturomics
Source: BMC Microbiol. 2019 Dec 30;19:308. doi: 10.1186/s12866-019-1681-6 (PMC6937742; doi:10.1186/s12866-019-1681-6)
Supplement: Supplementary file 1 — Additional file 1: Supplementary table and figure. [file 12866_2019_1681_MOESM1_ESM.pdf]

Journal name：*BMC Microbiology*

Manuscript Title： Application of LpxC enzyme inhibitor to inhibit some fast-growing bacteria in human gut bacterial culturomics

Running title： Application of inhibitor in gut bacterial culturomics

Author： Fengyi Hou[1,2#], Yuxiao Chang[2#], Zongyu Huang[2], Ni Han[2], Lei Bin[2], Huimin Deng[2], Zhengchao Li[2], Zhiyuan Pan[2], Lei Ding[3], Hong Gao[3], Ruifu Yang[2, a], Fachao Zhi[1*], Yujing Bi[2*]

[1] Guangdong Provincial Key Laboratory of Gastroenterology, Institute of Gastroenterology of Guangdong Province, Department of Gastroenterology, Nanfang Hospital, Southern Medical University, Guangzhou, China.

[2] State Key Laboratory of Pathogen and Biosecurity, Beijing Institute of Microbiology and Epidemiology, Beijing, China.

[3] Shijitan Hospital, Capital Medical University, Beijing, China.

[#]These authors contributed equally to this work.

[a]Senior author

*Corresponding authors: Yujing Bi (byj7801@sina.com) and Fachao Zhi (zhifc41532@163.com)

email addresses:

Fengyi Hou: hfy120356@163.com

Yuxiao Chang: changyuxiao3368@163.com

Zongyu Huang: 806057226@qq.com

Ni Han: 694025102@qq.com

Lei Bin: 624785188@qq.com

Huimin Deng: tammy054@163.com

Zhengchao Li: 506569665@qq.com

Zhiyuan Pan: 705088752@qq.com

Lei Ding: dinglei1005@126.com

Hong Gao: gaohongdoctor@sina.com

Ruifu Yang: 13801034560@163.com

Fachao Zhi: zhifc41532@163.com

Yujing Bi: byj7801@sina.com

Supplementary table & figure

Table S1 LpxC (UDP-3-O-acyl-N-acetylglucosamine deacetylase) coding sequence.

	Bacteria	LpxC source
Sensitive	*Escherichia coli*	ARTFGFMRDIEYLQSRGLCLGGSFDCAIVVDDYRVLN
	Pseudomonas aeruginosa	ARTFGFMRDIEYLRSQNLALGGSVENAIVVDENRVLN
	Klebsiella pneumoniae	ARTFGFMRDIEYLQSRGLCLGGSFDCAIVVDDYRVLN
	Proteus vulgaris	ARTFGFMRDIEYLQSKGLCLGGSFDCAIVVDDYRVLN
Insensitive	*Bacteroides vulgatus*	ARTFVFVREIEPLLGAGLIKGGDLDNAIVIYEKEMSQ

LpxC from *Bacteroides vulgatus* has 36% amino acid sequence identity to *E. coli* LpxC. The amino acids located in the inhibitor-binding site are responsible for affinity. By aligning the sequences of four CHIR-090-sensitive and one insensitive LpxCs, we found out one residue in Insert II hydrophobic passage is different between these two categories. Conserved hydrophobic residues are colored in red. The amino acids of key residues in the Insert II hydrophobic passage that would influence the affinity of CHIR-090 to LpxC are colored in orange (cf. [12]).

Table S2 taxonomic information of 102 species of bacteria.

（see addition file “Table S2 taxonomic information of 102 species of bacteria.xlsx”）

Supplementary Figure

Fig. S1 a

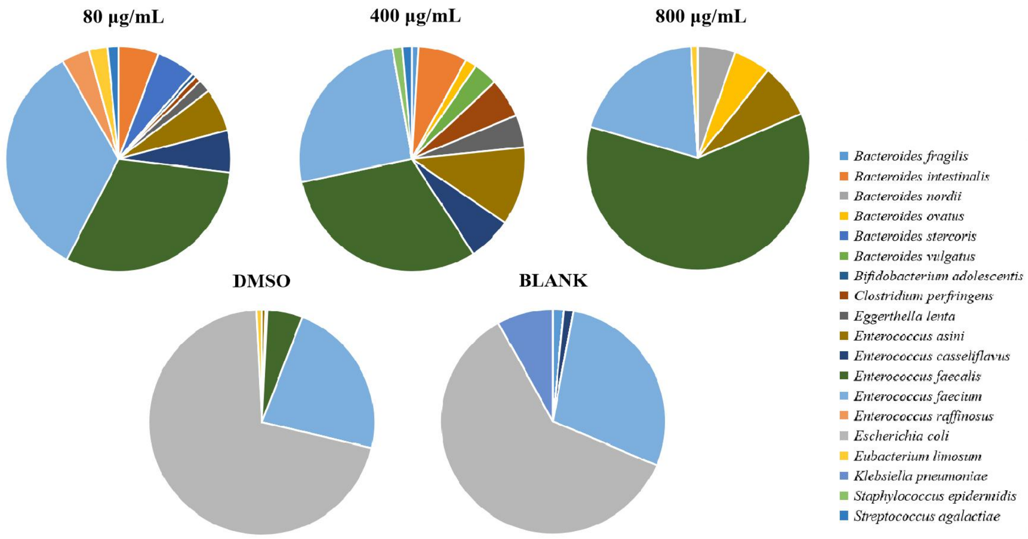

Fig. S1 b

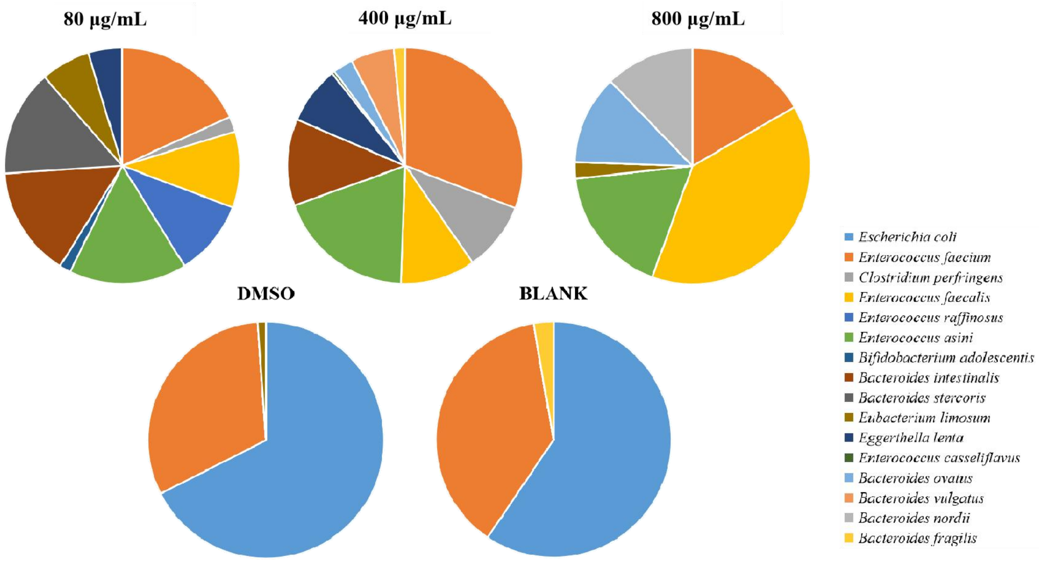

Fig. S1 c

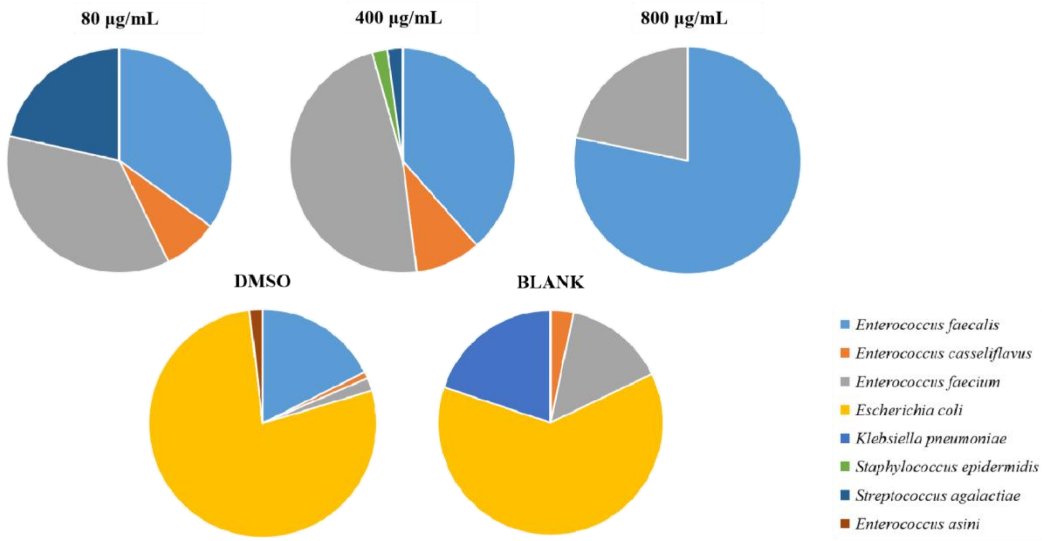

Fig. S1 a Percentages of colony-forming units (CFU) of bacteria of sample F1 cultured in with different concentrations of CHIR-090 under general condition (including anaerobic and aerobic concondition). In the DMSO and blank bottles (controls), *E. coli* and *Enterococcus faecium* occupied the largest percentages and only 6 and 5 species, respectively, of 19 species were isolated. In CHIR-090 bottles, *E. coli* was suppressed and the number of species increased to 12 in bottles with 80 and 400 μg/mL CHIR-090, and the species shared similar percentages. In bottles with 800

μg/mL CHIR-090, the diversity of species was reduced to 6 of 19 species and *Enterococcus faecalis* prevailed, although *E. coli* was not found. **b** Percentages of Colony-Forming Units (CFU) of bacteria after co-cultivating suspension of F1 with different concentration of CHIR-090 in anaerobic condition. **c** in aerobic condition. *E.coli* was totally suppressed in DMSO and BLANK bottles. CHIR-090 bottles of 80μg/mL and 400μg/mL showed the largest diversity of bacteria species while the diversity of 800μg/mL bottle are less.

Fig.S2a

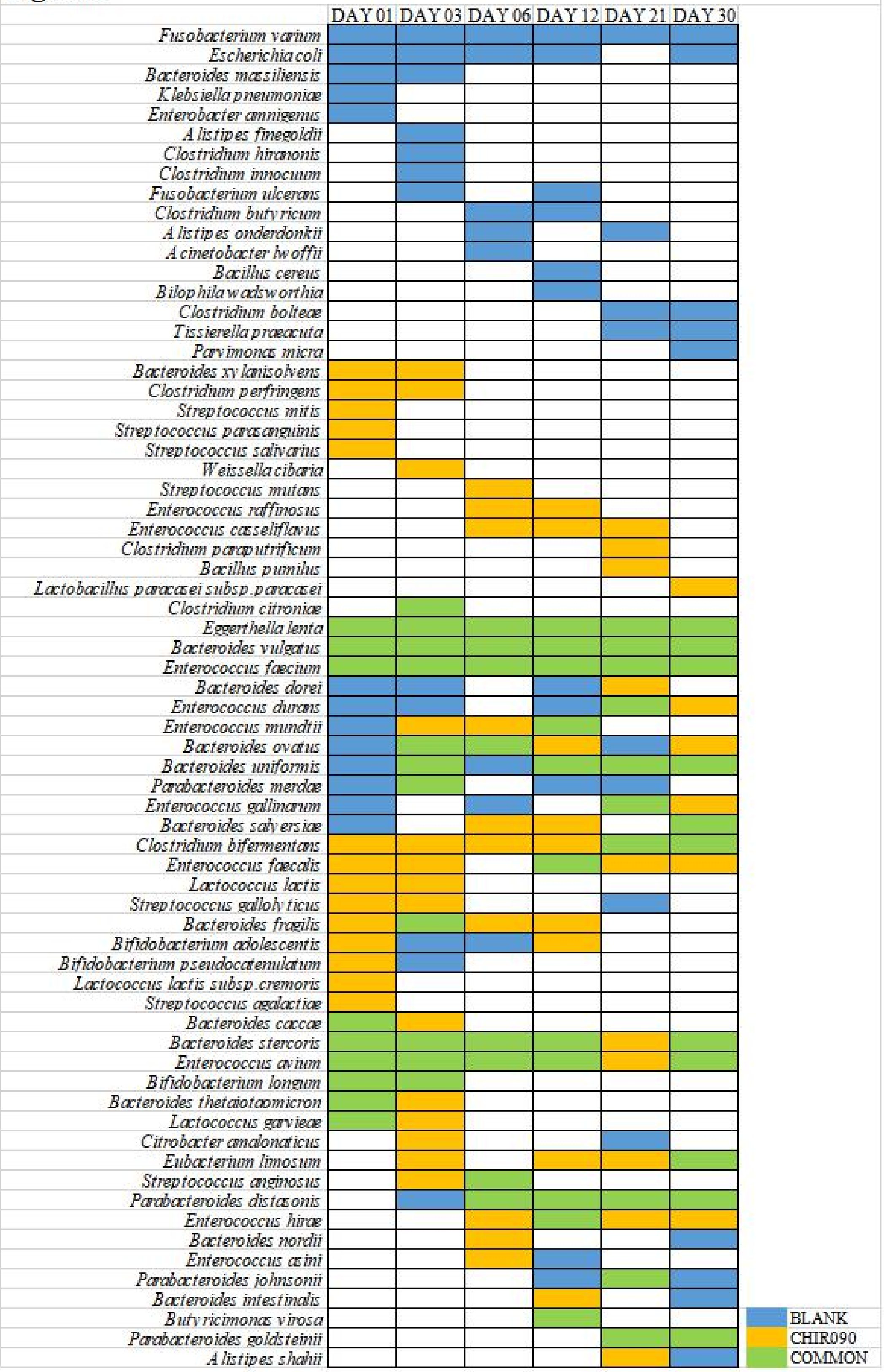
DAY 01
DAY 03
DAY 06
DAY 12
DAY 21
DAY 30
Fusobacterium varium
Escherichia coli
Bacteroides massiliensis
Klebsiella pneumoniae
Enterobacter amnigenus
Alistipes finegoldii
Clostridium hiranonis
Clostridium innocuum
Fusobacterium ulcerans
Clostridium butyricum
Alistipes onderdonkii
Acinetobacter lwoffii
Bacillus cereus
Bilophila wadsworthia
Clostridium bolteae
Tissierella praeacuta
Parvimonas micra
Bacteroides xylanisolvens
Clostridium perfringens
Streptococcus mitis
Streptococcus parasanguinis
Streptococcus salivarius
Weissella cibaria
Streptococcus mutans
Enterococcus raffinosus
Enterococcus casseliflavus
Clostridium paraputrificum
Bacillus pumilus
Lactobacillus paracasei subsp.paracasei
Clostridium citroniae
Eggerthella lenta
Bacteroides vulgatus
Enterococcus faecium
Bacteroides dorei
Enterococcus durans
Enterococcus mundtii
Bacteroides ovatus
Bacteroides uniformis
Parabacteroides merdae
Enterococcus gallinarum
Bacteroides salyersiae
Clostridium bifermentans
Enterococcus faecalis
Lactococcus lactis
Streptococcus gallolyticus
Bacteroides fragilis
Bifidobacterium adolescentis
Bifidobacterium pseudocatenulatum
Lactococcus lactis subsp.cremoris
Streptococcus agalactiae
Bacteroides caccae
Bacteroides stercoris
Enterococcus avium
Bifidobacterium longum
Bacteroides thetaiotaomicron
Lactococcus garvieae
Citrobacter amalonaticus
Eubacterium limosum
Streptococcus anginosus
Parabacteroides distasonis
Enterococcus hirae
Bacteroides nordii
Enterococcus asini
Parabacteroides johnsonii
Bacteroides intestinalis
Butyricimonas virosa
Parabacteroides goldsteinii
Alistipes shahii
BLANK
CHIR090
COMMON

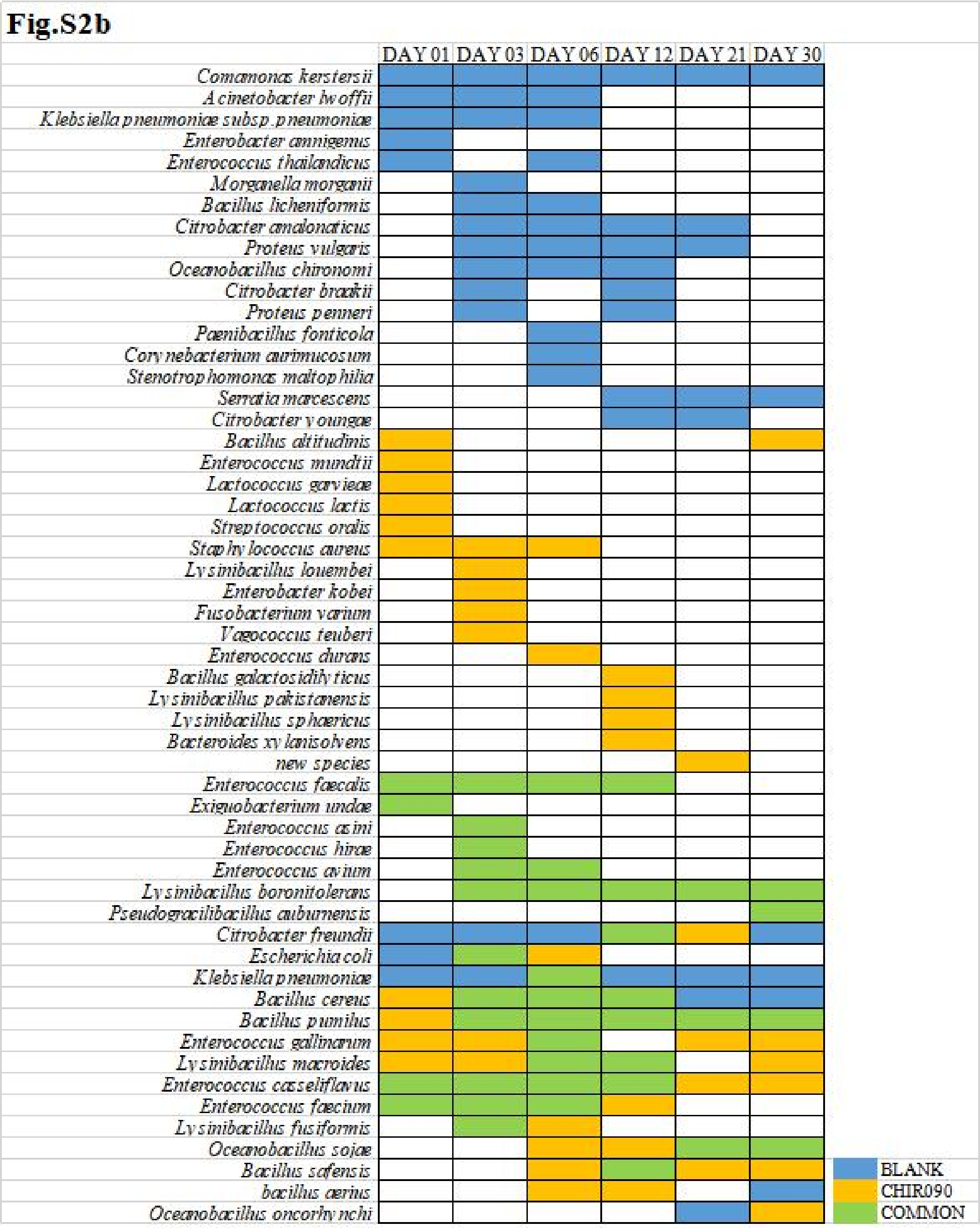

Fig. S2 **a** Distribution of all the anaerobic bacteria in 6 points in time. **b** Distribution of all the aerobic bacteria in 6 points in time.The blue color represents Blank group, the yellow represents CHIR-090 group, and the green represent existence in both bottles (COMMON group).

Fig.S3a

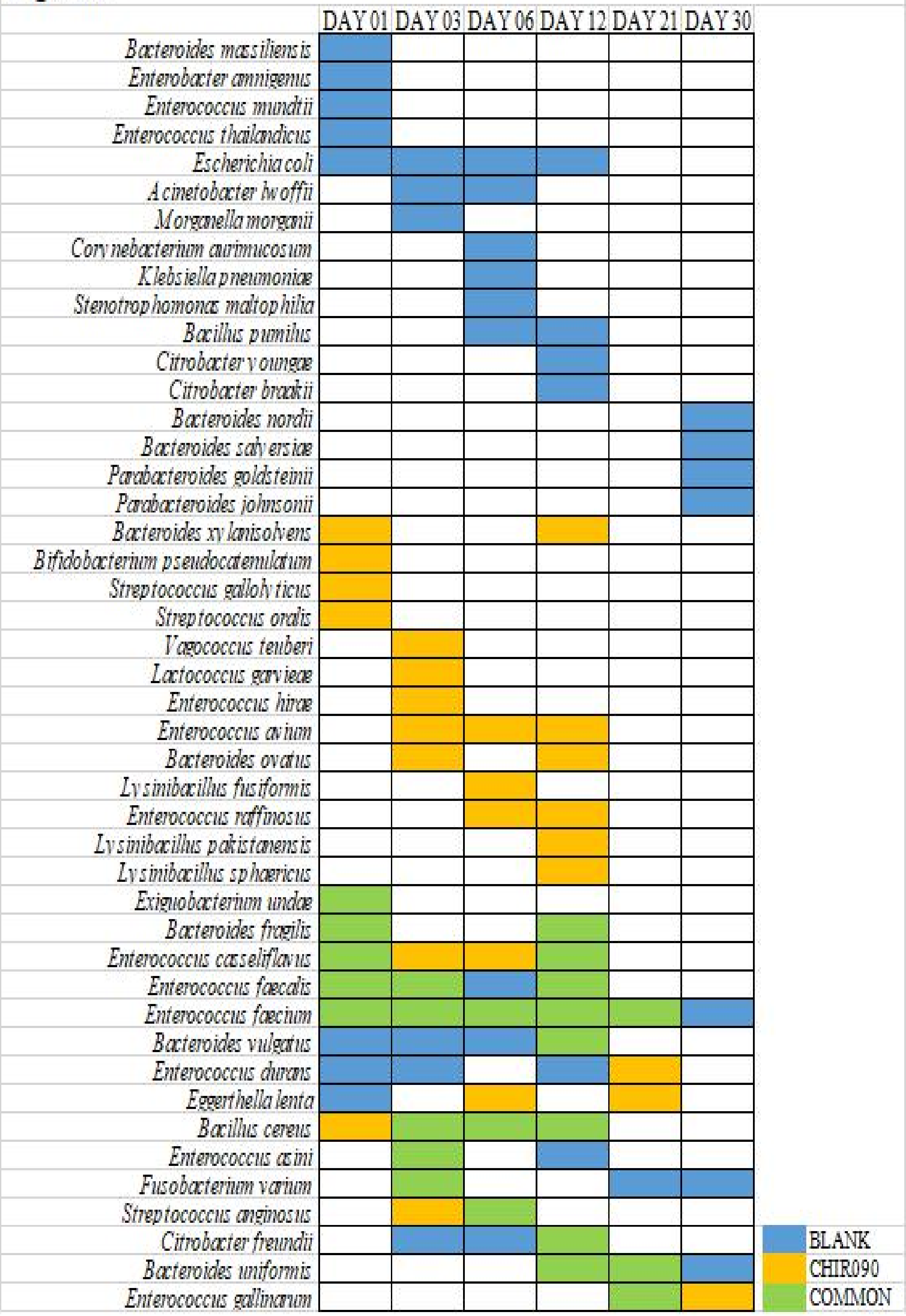
DAY 01
DAY 03
DAY 06
DAY 12
DAY 21
DAY 30
Bacteroides massiliensis
Enterobacter amnigenus
Enterococcus mundtii
Enterococcus thailandicus
Escherichia coli
Acinetobacter lwoffii
Morganella morganii
Corynebacterium aurimucosum
Klebsiella pneumoniae
Stenotrophomonas maltophilia
Bacillus pumilus
Citrobacter youngae
Citrobacter braakii
Bacteroides nordii
Bacteroides salyersiae
Parabacteroides goldsteinii
Parabacteroides johnsonii
Bacteroides xylanisolvens
Bifidobacterium pseudocatenulatum
Streptococcus gallolyticus
Streptococcus oralis
Vagococcus teuberi
Lactococcus garvieae
Enterococcus hirae
Enterococcus avium
Bacteroides ovatus
Lysinibacillus fusiformis
Enterococcus raffinosus
Lysinibacillus pakistanensis
Lysinibacillus sphaericus
Exiguobacterium undae
Bacteroides fragilis
Enterococcus casseliflavus
Enterococcus faecalis
Enterococcus faecium
Bacteroides vulgatus
Enterococcus durans
Eggerthella lenta
Bacillus cereus
Enterococcus asini
Fusobacterium varium
Streptococcus anginosus
Citrobacter freundii
Bacteroides uniformis
Enterococcus gallinarum
BLANK
CHIR090
COMMON

Fig.S3b

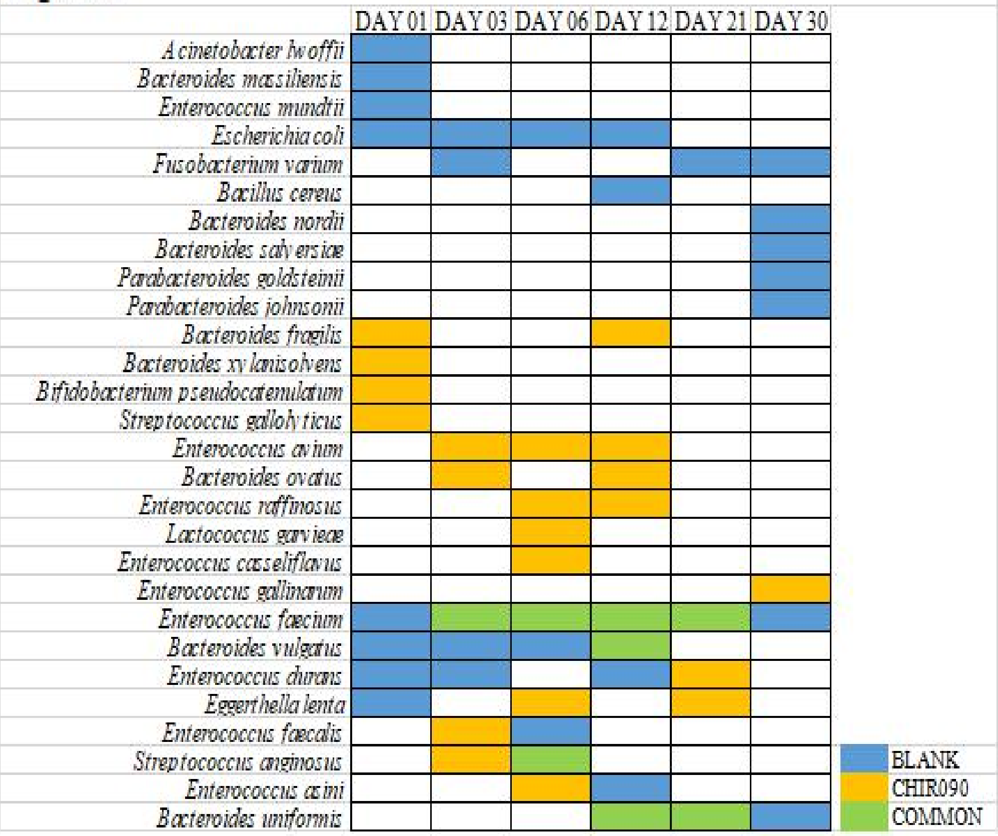

Fig.S3c

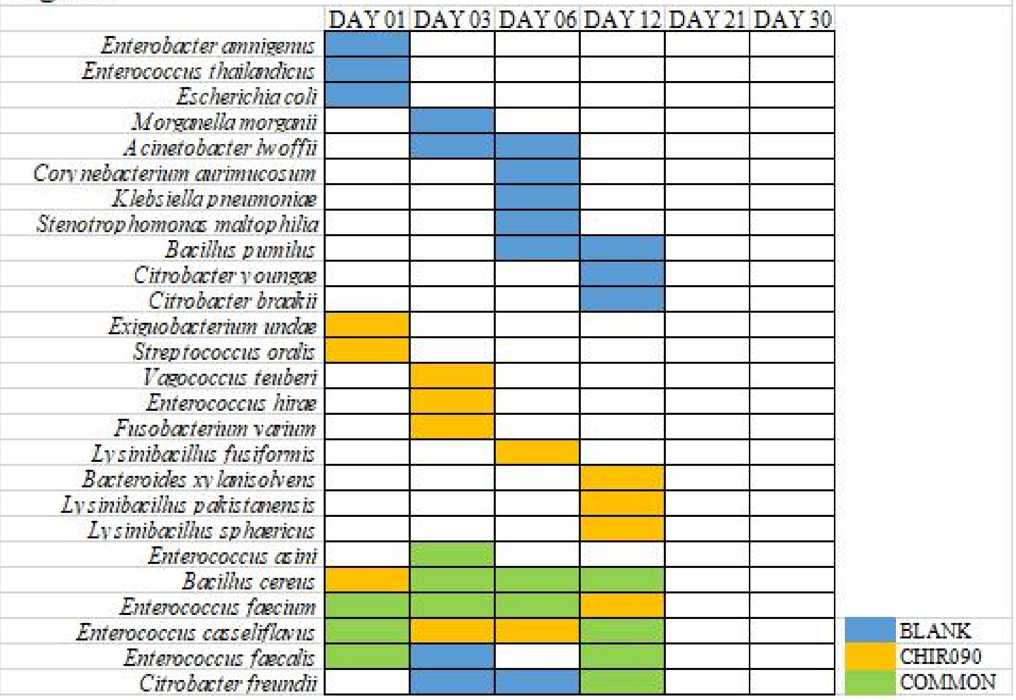

Fig.S3d

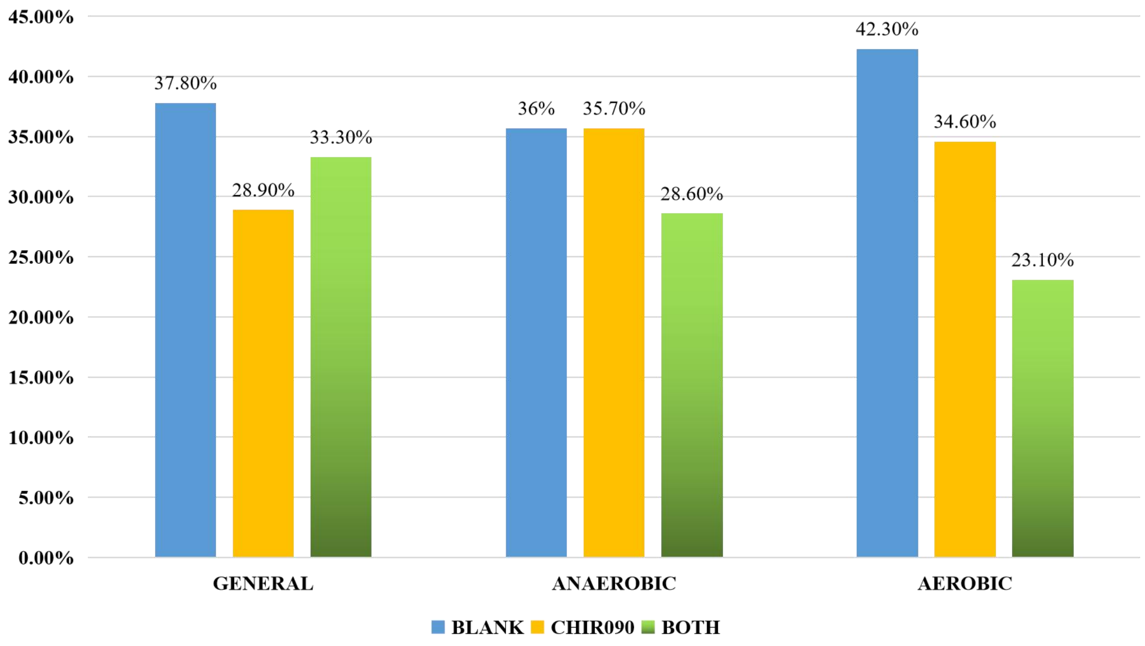
45.00%
40.00%
35.00%
30.00%
25.00%
20.00%
15.00%
10.00%
5.00%
0.00%
37.80%
28.90%
33.30%
36%
35.70%
28.60%
42.30%
34.60%
23.10%
GENERAL
ANAEROBIC
AEROBIC
BLANK
CHIR090
BOTH

Fig.S3e

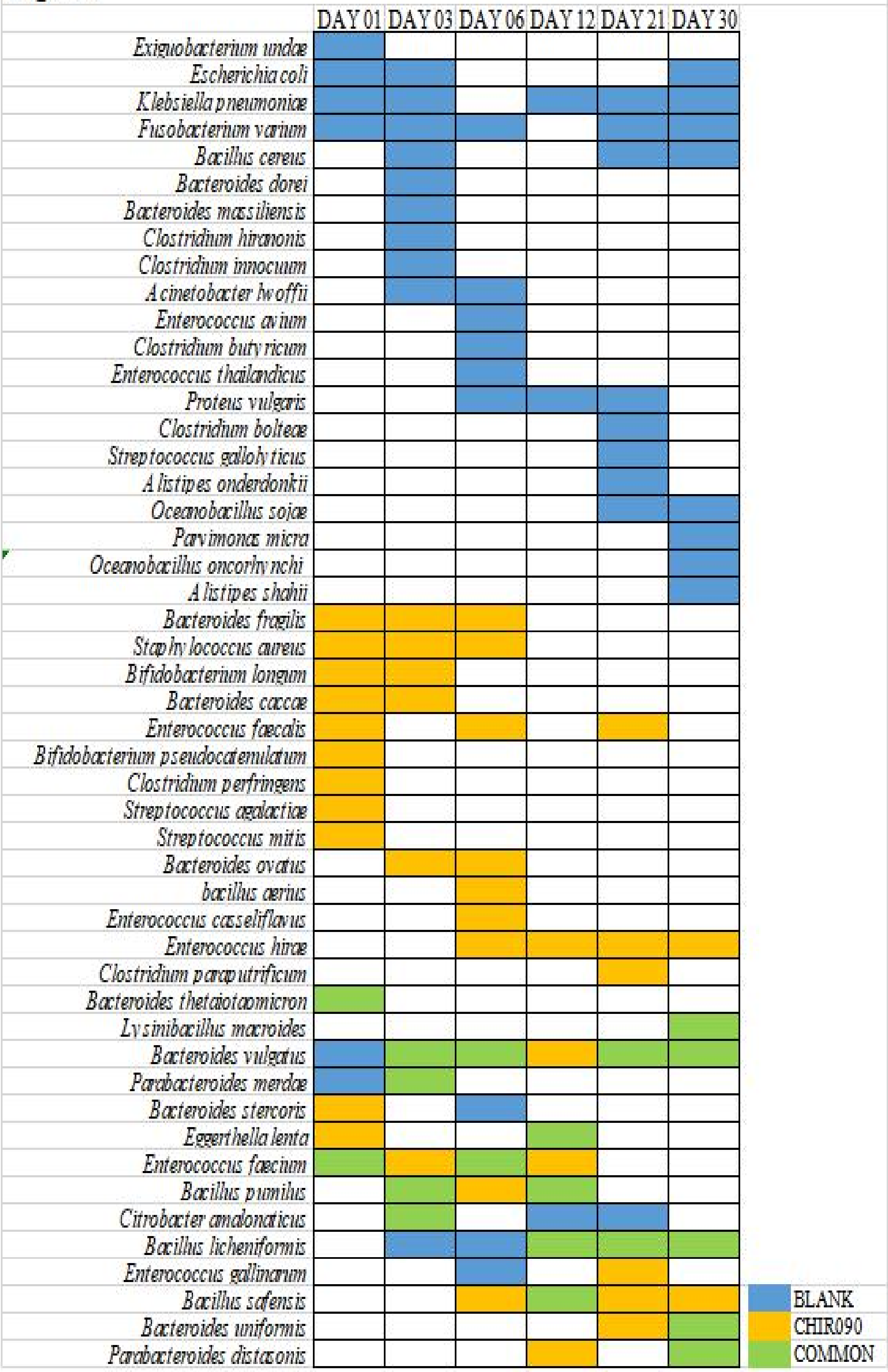
DAY 01
DAY 03
DAY 06
DAY 12
DAY 21
DAY 30
Exiguobacterium undae
Escherichia coli
Klebsiella pneumoniae
Fusobacterium varium
Bacillus cereus
Bacteroides dorei
Bacteroides massiliensis
Clostridium hiranonis
Clostridium innocuum
Acinetobacter lwoffii
Enterococcus avium
Clostridium butyricum
Enterococcus thailandicus
Proteus vulgaris
Clostridium bolteae
Streptococcus gallolyticus
Alistipes onderdonkii
Oceanobacillus sojae
Parvimonas micra
Oceanobacillus oncorhynchi
Alistipes shahii
Bacteroides fragilis
Staphylococcus aureus
Bifidobacterium longum
Bacteroides caccae
Enterococcus faecalis
Bifidobacterium pseudocatenulatum
Clostridium perfringens
Streptococcus agalactiae
Streptococcus mitis
Bacteroides ovatus
bacillus aerius
Enterococcus casseliflavus
Enterococcus hirae
Clostridium paraputrificum
Bacteroides thetaiotaomicron
Lysinibacillus macroides
Bacteroides vulgatus
Parabacteroides merdae
Bacteroides stercoris
Eggerthella lenta
Enterococcus faecium
Bacillus pumilus
Citrobacter amalonaticus
Bacillus licheniformis
Enterococcus gallinarum
Bacillus safensis
Bacteroides uniformis
Parabacteroides distasonis
BLANK
CHIR090
COMMON

Fig.S3f

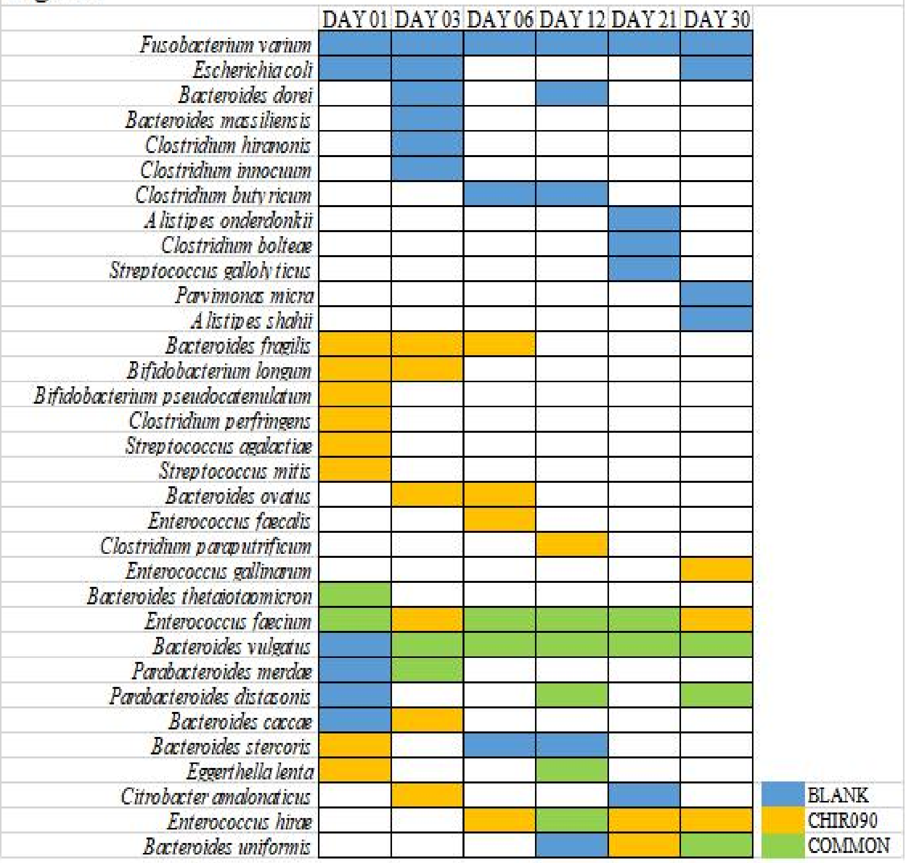

Fig.S3g

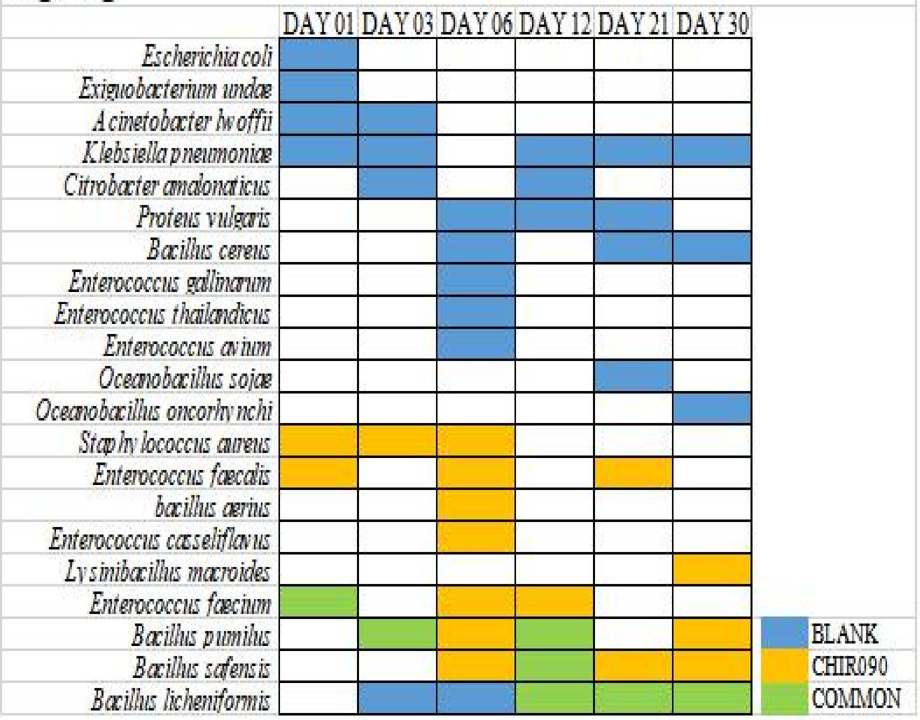

Fig. S3h

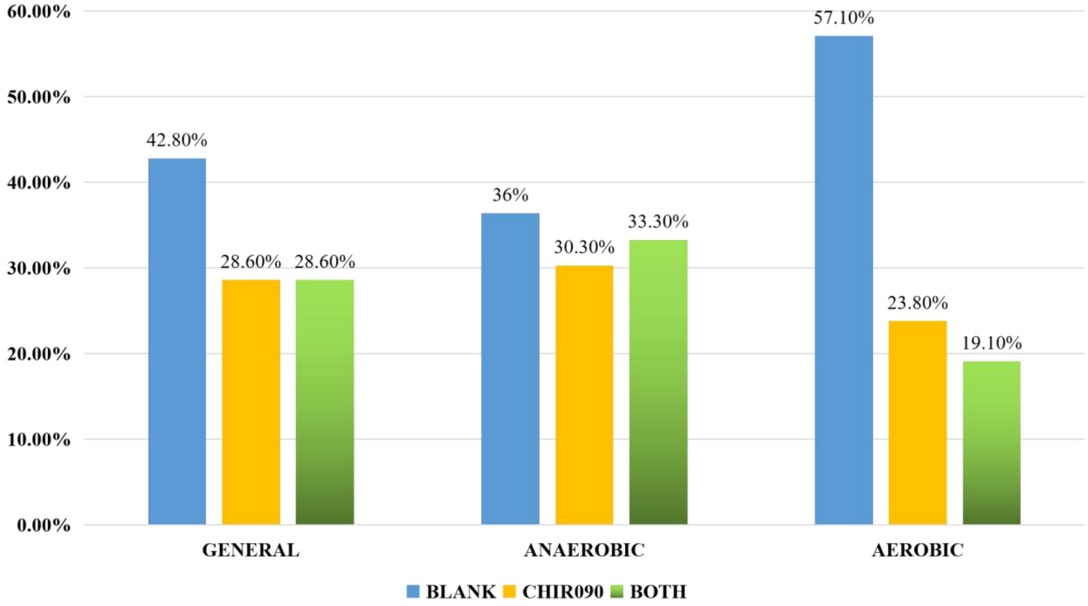
60.00%
50.00%
40.00%
30.00%
20.00%
10.00%
0.00%
42.80%
28.60%
28.60%
36%
30.30%
33.30%
57.10%
23.80%
19.10%
GENERAL
ANAEROBIC
AEROBIC
BLANK
CHIR090
BOTH

Fig.S3i

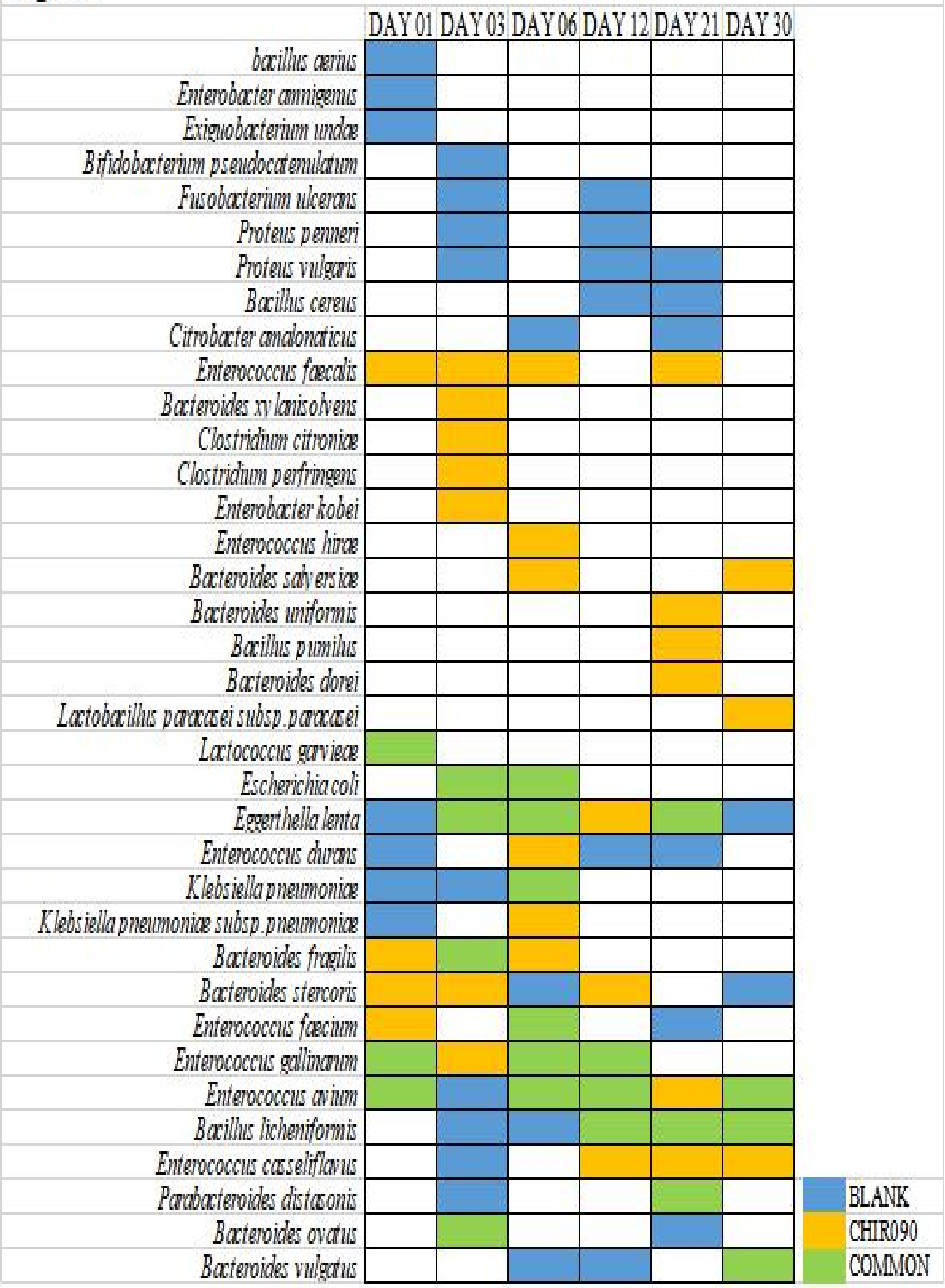
DAY 01
DAY 03
DAY 06
DAY 12
DAY 21
DAY 30
bacillus aerius
Enterobacter annigenus
Exiguobacterium undae
Bifidobacterium pseudocatenulatum
Fusobacterium ulcerans
Proteus penneri
Proteus vulgaris
Bacillus cereus
Citrobacter amalonaticus
Enterococcus faecalis
Bacteroides xylanisolvens
Clostridium citroniae
Clostridium perfringens
Enterobacter kobei
Enterococcus hirae
Bacteroides salyersiae
Bacteroides uniformis
Bacillus pumilus
Bacteroides dorei
Lactobacillus paracasei subsp.paracasei
Lactococcus garvieae
Escherichia coli
Eggerthella lenta
Enterococcus durans
Klebsiella pneumoniae
Klebsiella pneumoniae subsp.pneumoniae
Bacteroides fragilis
Bacteroides stercoris
Enterococcus faecium
Enterococcus gallinarum
Enterococcus avium
Bacillus licheniformis
Enterococcus casseliflavus
Parabacteroides distasonis
Bacteroides ovatus
Bacteroides vulgatus
BLANK
CHIR090
COMMON

Fig.S3j

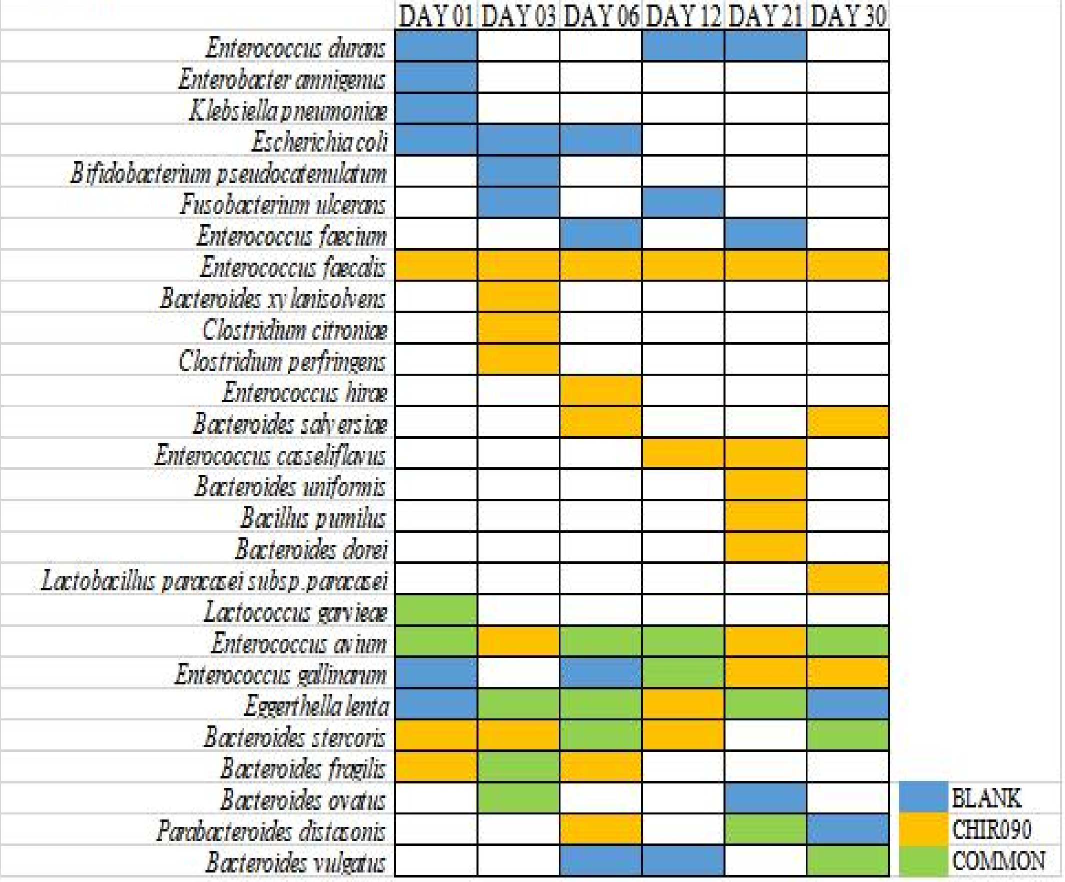

Fig.S3k

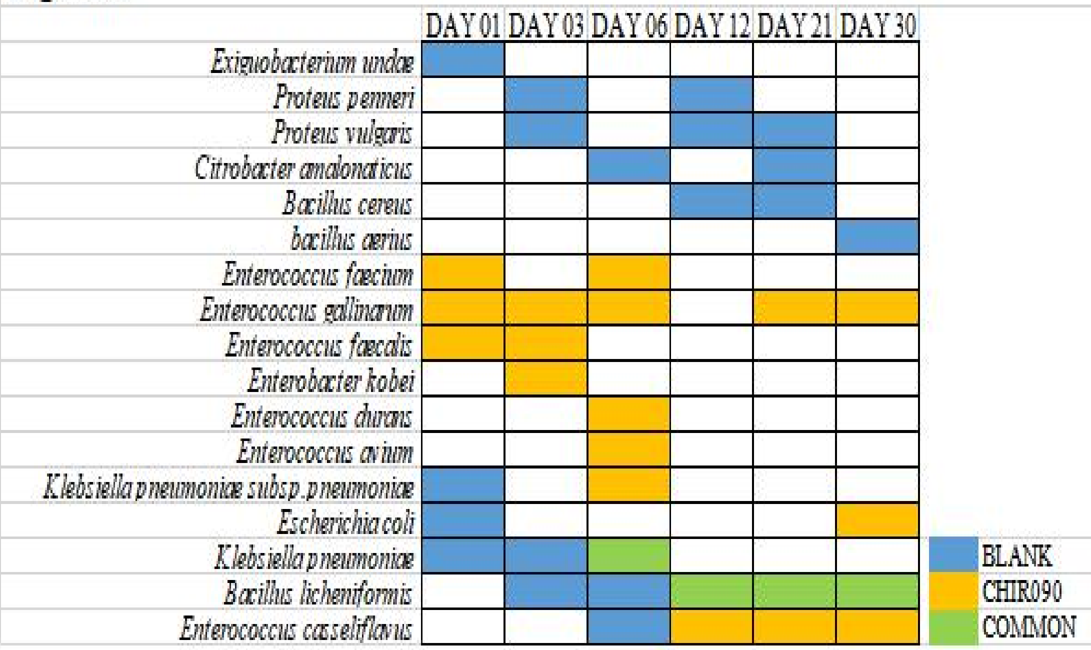

Fig. S3l

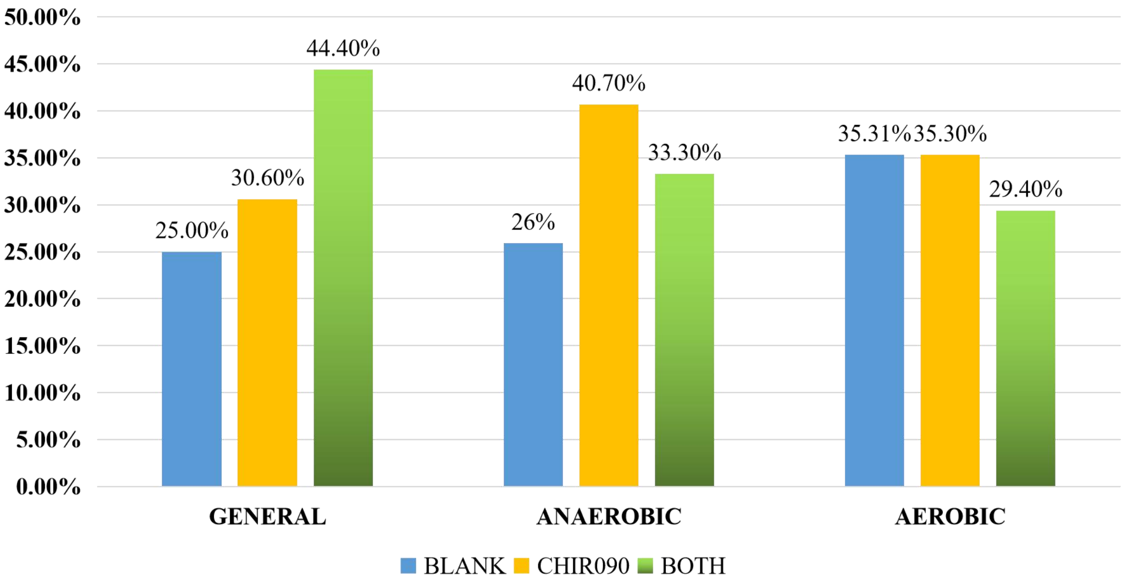
50.00%
45.00%
40.00%
35.00%
30.00%
25.00%
20.00%
15.00%
10.00%
5.00%
0.00%
25.00%
30.60%
44.40%
26%
40.70%
33.30%
35.31%
35.30%
29.40%
GENERAL
ANAEROBIC
AEROBIC
BLANK
CHIR090
BOTH

Fig.S3m

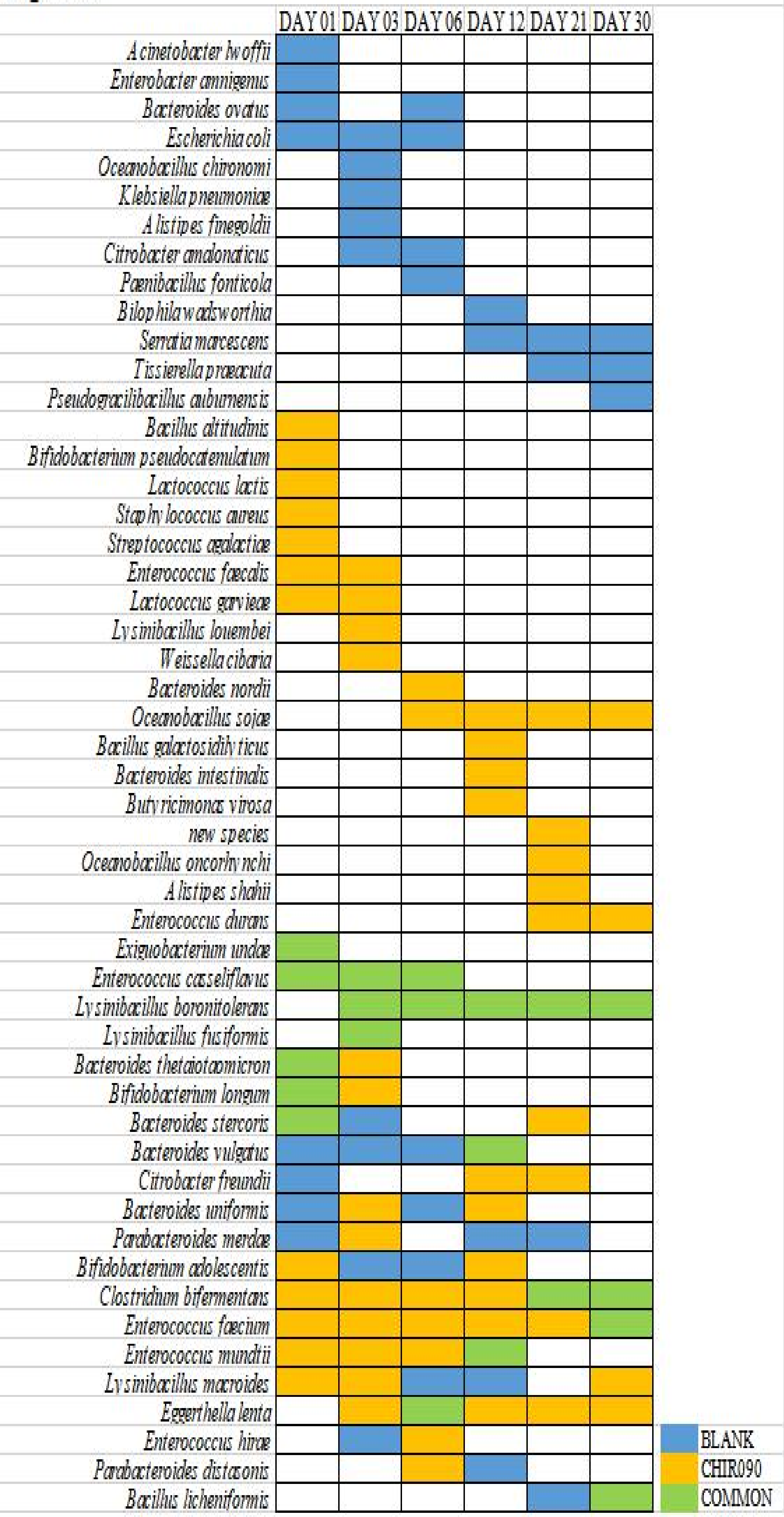
DAY 01
DAY 03
DAY 06
DAY 12
DAY 21
DAY 30
Acinetobacter lwoffii
Enterobacter amnigenus
Bacteroides ovatus
Escherichia coli
Oceanobacillus chironomi
Klebsiella pneumoniae
Alistipes finegoldii
Citrobacter amalonaticus
Paenibacillus fonticola
Bilophila wadsworthia
Serratia marcescens
Tissierella praeacuta
Pseudogracilibacillus auburnensis
Bacillus altitudinis
Bifidobacterium pseudocatenulatum
Lactococcus lactis
Staphylococcus aureus
Streptococcus agalactiae
Enterococcus faecalis
Lactococcus garvieae
Lysinibacillus louembei
Weissella cibaria
Bacteroides nordii
Oceanobacillus sojae
Bacillus galactosidilyticus
Bacteroides intestinalis
Butyricimonas virosa
new species
Oceanobacillus oncorhynchi
Alistipes shahii
Enterococcus durans
Exiguobacterium undae
Enterococcus casseliflavus
Lysinibacillus boronitolerans
Lysinibacillus fusiformis
Bacteroides thetaiotaomicron
Bifidobacterium longum
Bacteroides stercoris
Bacteroides vulgatus
Citrobacter freundii
Bacteroides uniformis
Parabacteroides merdae
Bifidobacterium adolescentis
Clostridium bifermentans
Enterococcus faecium
Enterococcus mundtii
Lysinibacillus macroides
Eggerthella lenta
Enterococcus hirae
Parabacteroides distasonis
Bacillus licheniformis
BLANK
CHIR090
COMMON

Fig.S3n

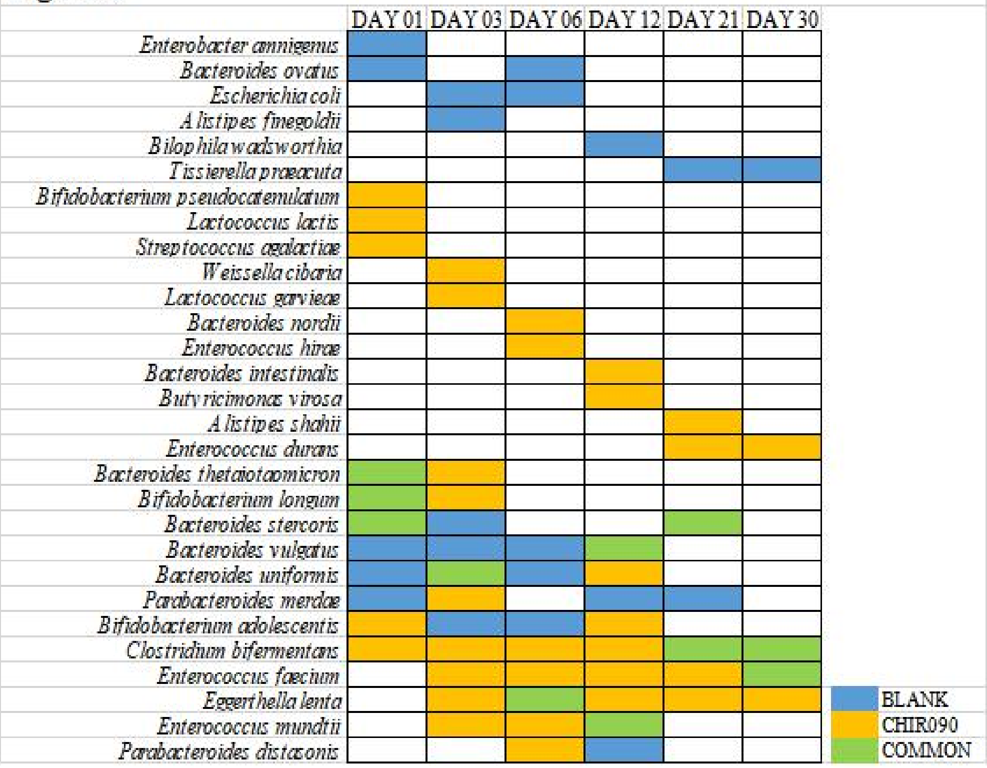

Fig.S3o

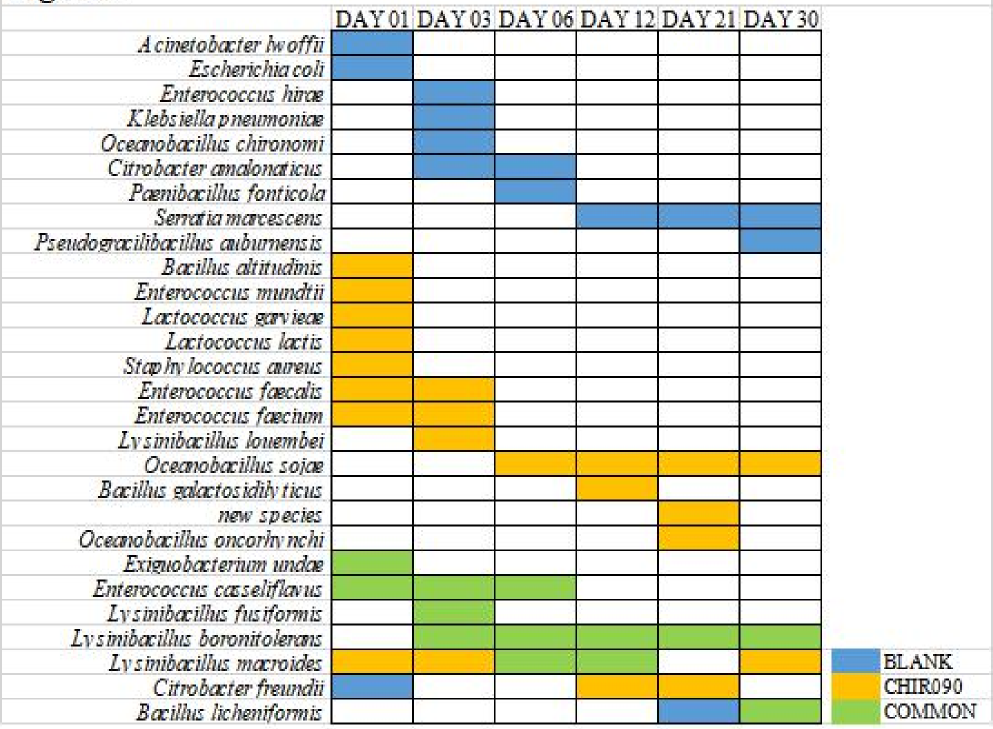

Fig. S3p

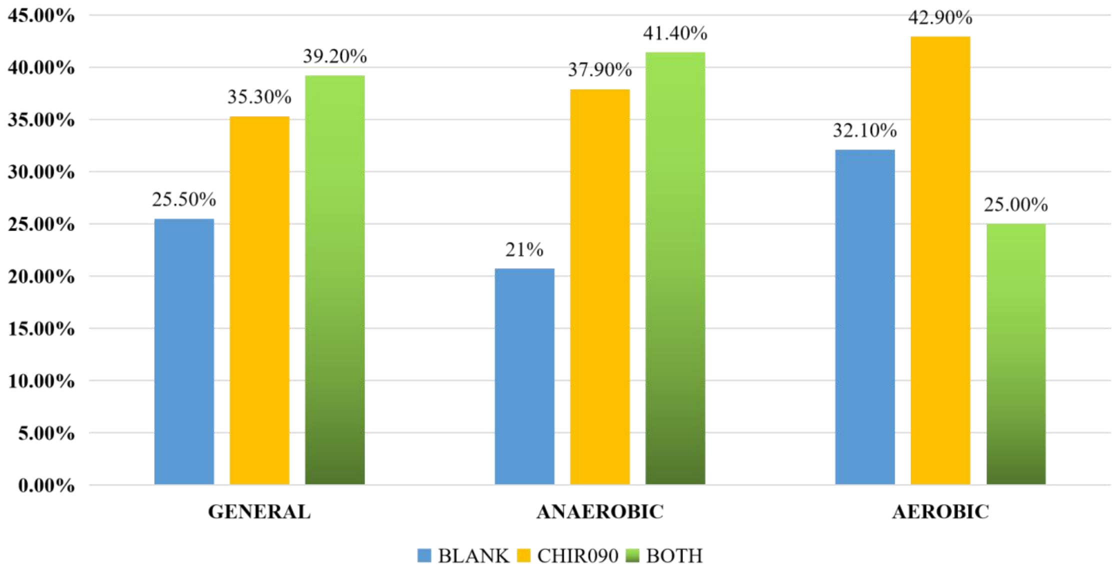
45.00%
40.00%
35.00%
30.00%
25.00%
20.00%
15.00%
10.00%
5.00%
0.00%
25.50%
35.30%
39.20%
21%
37.90%
41.40%
32.10%
42.90%
25.00%
GENERAL
ANAEROBIC
AEROBIC
BLANK
CHIR090
BOTH

Fig.S3q

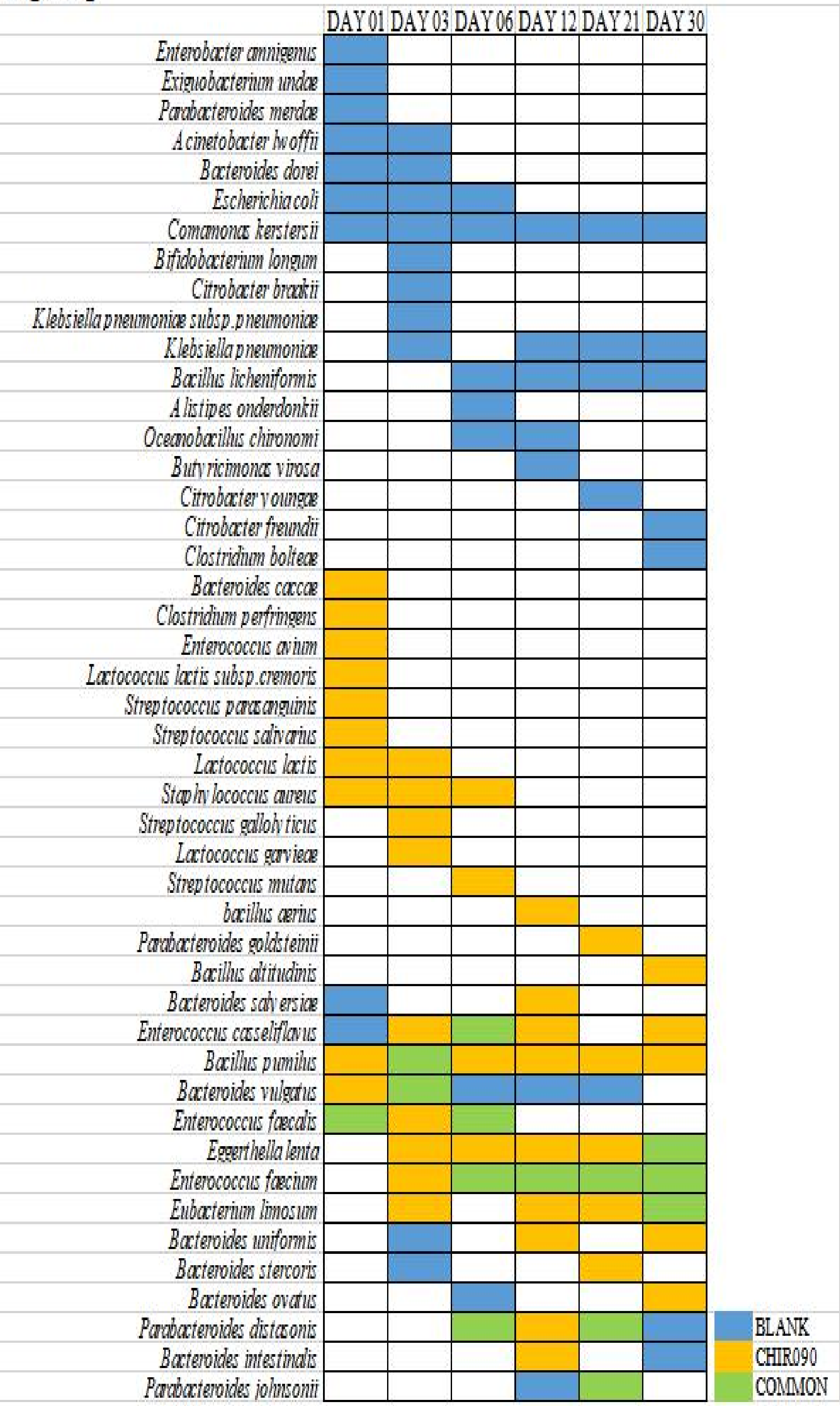
DAY 01
DAY 03
DAY 06
DAY 12
DAY 21
DAY 30
Enterobacter amnigenus
Exiguobacterium undae
Parabacteroides merdae
Acinetobacter lwoffii
Bacteroides dorei
Escherichia coli
Comamonas kerstersii
Bifidobacterium longum
Citrobacter braakii
Klebsiella pneumoniae subsp.pneumoniae
Klebsiella pneumoniae
Bacillus licheniformis
Alistipes onderdonkii
Oceanobacillus chironomi
Butyricimonas virosa
Citrobacter youngae
Citrobacter freundii
Clostridium bolteae
Bacteroides caccae
Clostridium perfringens
Enterococcus avium
Lactococcus lactis subsp.cremoris
Streptococcus parasanguinis
Streptococcus salivarius
Lactococcus lactis
Staphylococcus aureus
Streptococcus gallolyticus
Lactococcus garvieae
Streptococcus mutans
bacillus aerius
Parabacteroides goldsteinii
Bacillus altitudinis
Bacteroides salyersiae
Enterococcus casseliflavus
Bacillus pumilus
Bacteroides vulgatus
Enterococcus faecalis
Eggerthella lenta
Enterococcus faecium
Eubacterium limosum
Bacteroides uniformis
Bacteroides stercoris
Bacteroides ovatus
Parabacteroides distasonis
Bacteroides intestinalis
Parabacteroides johnsonii
BLANK
CHIR090
COMMON

Fig.S3r

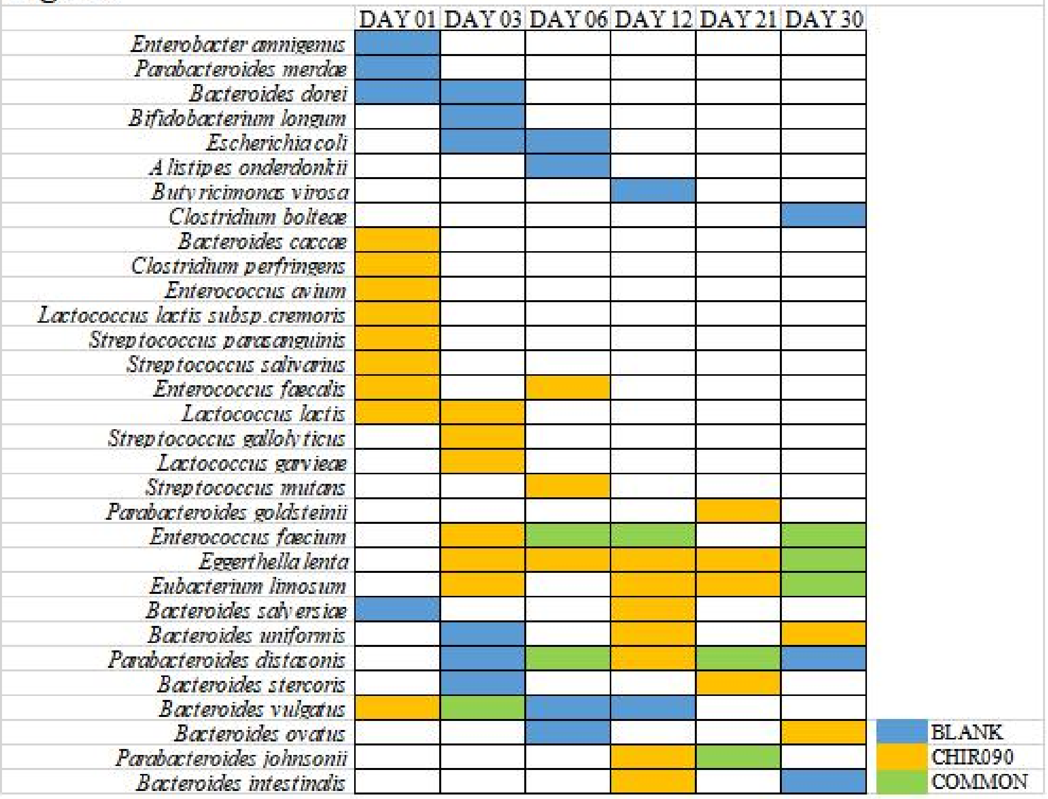

Fig.S3s

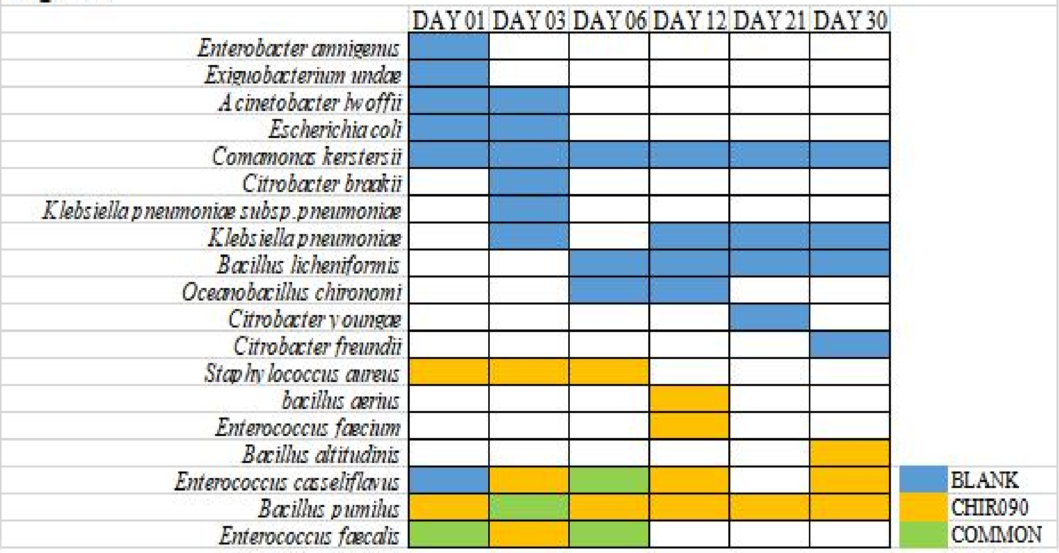

Fig. S3t

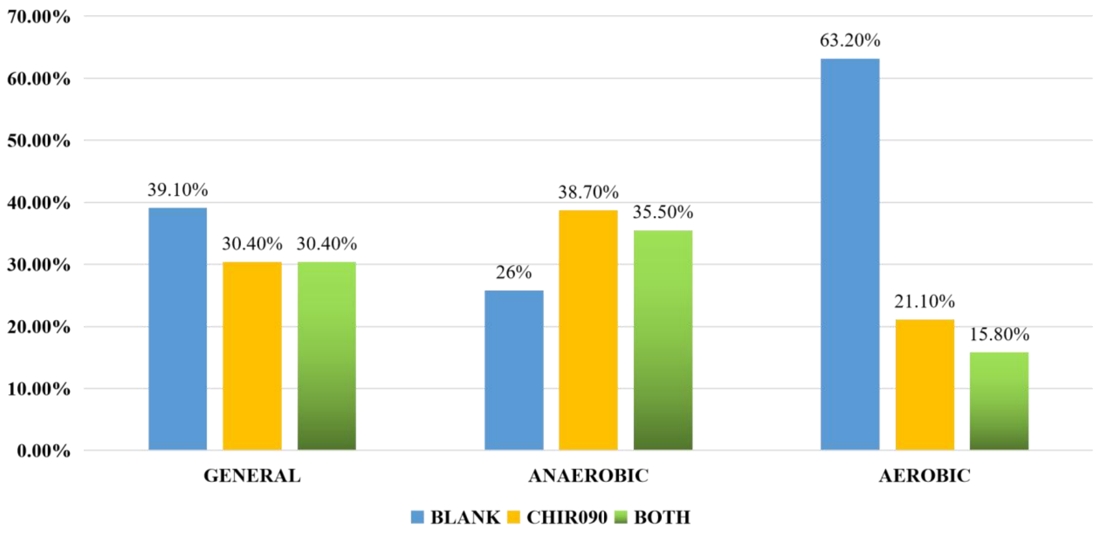

Fig. S3a The ditrubution of **F2** bacteria at 6 points in time in the general condition. **b** In anaerobic condition. **c** In aerobic condition. **d** The percentage of bacteria existed both/ respectively in BLANK, CHIR090 group in general, anaerobic and aerobic condition. **e** The ditrubution of **F3** bacteria at 6 points in time in the general condition. **f** In anaerobic condition. **g** In aerobic condition. **h** The percentage of bacteria existed both/ respectively in BLANK, CHIR090 group in general, anaerobic and aerobic condition. **i** The ditrubution of **F4** bacteria at 6 points in time in the general condition. **j** In anaerobic condition. **k** In aerobic condition. **l** The percentage of bacteria existed both/ respectively in BLANK, CHIR090 group in general, anaerobic and aerobic condition. **m** The ditrubution of **F5** bacteria at 6 points in time in the general condition. **n** In anaerobic condition. **o** In aerobic condition. **p** The percentage of bacteria existed both/ respectively in BLANK, CHIR090 group in general, anaerobic and aerobic condition. **q** The ditrubution of **F6** bacteria at 6 points in time in the general condition. **r** In anaerobic condition. **s** In aerobic condition. **t** The percentage

of bacteria existed both/ respectively in BLANK, CHIR090 group in general, anaerobic and aerobic condition. The blue color represents Blank group, the yellow represents CHIR-090 group, and the green represent existence in both bottles (COMMON group).
